# The Role of Cysteine Residues in Redox Regulation and Protein Stability of *Arabidopsis thaliana* Starch Synthase 1

**DOI:** 10.1371/journal.pone.0136997

**Published:** 2015-09-14

**Authors:** Katsiaryna Skryhan, Jose A. Cuesta-Seijo, Morten M. Nielsen, Lucia Marri, Silas B. Mellor, Mikkel A. Glaring, Poul E. Jensen, Monica M. Palcic, Andreas Blennow

**Affiliations:** 1 Copenhagen Plant Science Centre, Department of Plant and Environmental Sciences, University of Copenhagen, Thorvaldsensvej 40, 1871 Frederiksberg C, Denmark; 2 Carlsberg Laboratory, Gamle Carlsberg Vej 10, 1799 Copenhagen V, Denmark; 3 Department of Biochemistry and Microbiology, University of Victoria, Victoria, British Columbia, Canada; Instituto de Biociencias - Universidade de São Paulo, BRAZIL

## Abstract

Starch biosynthesis in *Arabidopsis thaliana* is strictly regulated. In leaf extracts, starch synthase 1 (*At*SS1) responds to the redox potential within a physiologically relevant range. This study presents data testing two main hypotheses: 1) that specific thiol-disulfide exchange in *At*SS1 influences its catalytic function 2) that each conserved Cys residue has an impact on *At*SS1 catalysis. Recombinant *At*SS1 versions carrying combinations of cysteine-to-serine substitutions were generated and characterized *in vitro*. The results demonstrate that *At*SS1 is activated and deactivated by the physiological redox transmitters thioredoxin *f*1 (Trx*f*1), thioredoxin *m*4 (Trx*m*4) and the bifunctional NADPH-dependent thioredoxin reductase C (NTRC). *At*SS1 displayed an activity change within the physiologically relevant redox range, with a midpoint potential equal to -306 mV, suggesting that *At*SS1 is in the reduced and active form during the day with active photosynthesis. Cys164 and Cys545 were the key cysteine residues involved in regulatory disulfide formation upon oxidation. A C164S_C545S double mutant had considerably decreased redox sensitivity as compared to wild type *At*SS1 (30% vs 77%). Michaelis-Menten kinetics and molecular modeling suggest that both cysteines play important roles in enzyme catalysis, namely, Cys545 is involved in ADP-glucose binding and Cys164 is involved in acceptor binding. All the other single mutants had essentially complete redox sensitivity (98–99%). In addition of being part of a redox directed activity “light switch”, reactivation tests and low heterologous expression levels indicate that specific cysteine residues might play additional roles. Specifically, Cys265 in combination with Cys164 can be involved in proper protein folding or/and stabilization of translated protein prior to its transport into the plastid. Cys442 can play an important role in enzyme stability upon oxidation. The physiological and phylogenetic relevance of these findings is discussed.

## Introduction

Starch is a major storage carbohydrate in plants, a main source of nutrition in the human and animal diet as well as a biorenewable and biodegradable material with a wide range of non-food applications [1]. Starch is composed exclusively of two structurally distinct α-D-glucose polymers, amylose and amylopectin, and is stored in the form of water-insoluble semi-crystalline granules. Amylose typically accounts for 10–30% of starch weight and is a mostly linear polymer. It consists of a wide distribution of chain lengths of approximately 1000 glucose units joined by α-(1→4)-glucosidic bonds with a low frequency (<1%) of branch points provided by α-(1→6)-glucosidic bonds. Amylopectin, which typically accounts for 70–90% of starch weight, is a moderately branched macromolecule (5–6%) consisting of 10^5^−10^6^ glucose units. Amylopectin glucose residues are linked by α-(1→4)-bonds and via branching α-(1→6)-bonds.

Starch is of fundamental importance for plant survival and adaptation to varying environmental conditions. Starch granules are synthesized and stored inside plastids, mainly chloroplasts in the photosynthetically active tissues (transient starch) and amyloplasts in storage organs such as tubers, grains and roots (storage starch). Transient starch is produced during the day and is degraded during the following night to support continuous plant growth and nocturnal metabolism. Storage starch is synthesized from imported sucrose and is metabolized during seed germination, sprouting or stress response processes [2]. Mutants impaired in transient starch accumulation reveal reduced growth rates under various environmental conditions [3].

Transient starch is produced by the coordinated action of ADP-glucose pyrophosphorylase (AGPase), starch synthases (SSs), branching enzymes (BEs), debranching enzymes (DBEs) and glucan-water dikinases (GWDs) [4]. AGPase generates the activated glucosyl donor ADP-glucose which is used by SSs to elongate chains of amylose and amylopectin by adding the glucose moiety exclusively to the non-reducing ends (NREs). BEs create α-(1→6)-branches, increasing the number of NREs and thus facilitating SSs activity. DBEs remove excessive branches to form the crystalline structure. During biosynthesis, starch phosphorylation provided by GWDs modulates starch crystallinity and prepares the granule for solubilization prior to degradation [5].

Starch metabolism is tightly regulated at many levels including allosteric regulation by metabolites, protein-protein interactions, protein phosphorylation and redox regulation [6]. The redox state of the plant photosynthetic cell integrates a variety of metabolic processes, including transient starch turnover, with the availability of energy, reducing equivalents and assimilated carbon generated during the day [7]. The main source of reducing power in the plant cell is the linear electron transport in photosynthesis, which occurs in chloroplasts. Ferredoxin (Fdx) receives electrons from photosystem I (PSI) and then interacts with various enzymes to directly mediate reduction of NADP^+^ to NADPH, reduction of thioredoxins (Trxs) and other processes, such as reduction of sulfite and nitrite [8–10]. NADPH and Trxs are subsequently used in biosynthetic processes, such as the Calvin-Benson cycle and fatty acid and chlorophyll biosynthesis, and in antioxidant reactions and redox regulation of target proteins [11].

Thioredoxins are ubiquitous small proteins (ca. 12 kDa) belonging to the oxidoreductase family of enzymes and play a fundamental role in regulating multiple cellular processes [12,13]. They catalyze thiol-disulfide exchange in their target proteins, which currently number close to 500 in oxygenic photosynthetic organisms [14]. The active site of Trxs is located within the characteristic thioredoxin fold and contains two reactive cysteine (Cys) residues in a conserved CXXC motif. Unlike bacteria and animals, plants contain a large number of Trxs with different localizations, which serve a broad range of cellular functions. Recently the importance of Trx*f* for starch accumulation was shown *in planta* using tobacco plants overexpressing the Trx*f* [15] and Arabidopsis mutants lacking Trx*f1* [16].

NADPH-dependent thioredoxin reductase C (NTRC) is a recently discovered plastidic enzyme contributing to the antioxidant system as well as to regulation of carbohydrate metabolism. Initially NTRC was discovered as a light-independent reductant of 2-cysteine peroxiredoxin (2-Cys-Prx). Because of its ability to use NADPH generated via the oxidative pentose phosphate pathway (OPP) at night, NTRC is thought to provide an efficient antioxidant defense at a time when Trxs are likely to be oxidized [17]. NTRC was also demonstrated to promote starch accumulation in response to light or external sucrose treatment via redox-dependent AGPase activation [18].

During photosynthesis, chloroplast-localized Trxs and NTRC are reduced via Fdx/Trx reductase (FTR) and NADPH generated by Fdx-NADP reductase (FNR), respectively. In chloroplasts at night and in amyloplasts, NADPH generated via OPP could serve as a direct reductant for NTRC, which was shown to be present also in non-photosynthetic plastids [19] or via the FNR/FTR system for Trx [20]. In heterotrophic tissues this might play a crucial role in adjusting the starch accumulation rate in amyloplast to the sucrose transported from source tissues. Other cellular compartments also use NADPH to reduce Trx, but do so exclusively via the NADPH-dependent Trx reductases [21].

In starch biosynthesis, redox-dependent regulation involving key Cys residues has been identified in AGPase [22,23] and in GWD1 [24]. Using activity screening, a number of potential targets for redox-mediated post-translation modification among starch biosynthetic enzymes was recently identified, namely SS1, SS3, BE2 and ISA1/2 [25].

Starch synthase 1 from *Arabidopsis thaliana* (*At*SS1), which belong to the CAZY glycosyltransferase family 5 (GT5), is a key transferase in starch biosynthesis. It has a structural fold termed GT-B and a retaining mechanism of glucose transfer giving an α-configuration of the growing polyglucan chain (http://www.cazy.org/).

The aim of the current work was to identify the redox-active Cys residues involved in regulatory thiol-disulfide exchange reactions for *At*SS1 and to elucidate possible additional roles of conserved Cys residues for *At*SS1 functioning. Using recombinant *At*SS1 protein and its mutated versions generated by a site-directed mutagenesis approach, we identified a specific regulatory disulfide, which was related to catalytic activity. For *At*SS1 activity analysis two methods were utilised: spectrophotometric coupled glycosyltransferase assay (SCGA, adapted from [26]), and native activity gel electrophoresis with glycogen as a substrate (NAG, also called a zymogram, [27]).

Our data suggest distinct physiological roles of redox-active Cys residues in *At*SS1 including diurnal activity regulation directly linked to photosynthesis as well as stress-related protection of *At*SS1. On the basis of an evolutional analysis we suggest that this mechanism can be widespread in higher organisms to provide precise modulation of activity related to specific redox cues.

## Results

### The activity of *At*SS1 is redox sensitive and reversible

Our previous investigation [25] demonstrated that *At*SS1 in leaf extracts is reversibly sensitive to the redox potential in the medium. We confirmed that also the recombinant *At*SS1 used in the present work both displayed redox sensitivity as well as redox reversibility at the same levels as the enzyme in leaf extract (S1 Fig).

### Both enzyme activity and redox sensitivity are substrate dependent

*At*SS1 activity is dependent on acceptor substrate type [28]. We tested if redox sensitivity—defined as a percentage difference between reduced (set as 100%) and oxidized activity—is dependent on the nature of the glucan substrate using a linear series of maltooligosaccharides (MOS) from glucose to maltooctaose and glycogen as substrates. Under reducing conditions, the highest activity was observed with glycogen as a substrate (Table 1). The higher activity on glycogen could be explained by a higher enzyme affinity for glycogen than for MOS. MOS-surface binding sites found on the surface of N-terminal [29] and C-terminal Rossman folds (RFs) [30] could contribute to bridge and co-localise long branched substrates (*e*.*g*. amylopectin) with SS1 enzyme molecules, thus stabilizing binding and locally increasing the acceptor concentration in the active site of *At*SS1. The redox sensitivity values were very similar for all MOS (83% in average) while redox sensitivity for glycogen was markedly higher (96%).

**Table 1:** Determination of the glucan primer preference of *At*SS1. *At*SS1 protein was treated with either 20 mM DTTred or 20 mM DTTox to promote protein reduction and oxidation, respectively. All acceptors were used in 10 mM concentration except for glycogen which was 1 mg mL^-1^. The activity was assayed by SCGA. Results are the mean of two independent experiments (±SD) and are expressed as turnovers of enzyme per minute.

Acceptor type	Reduced *At*SS1		Oxidized *At*SS1	
	Enzyme activity A_red_	Activity relative to maltotriose (%)	Enzyme activity A_ox_	Redox sensitivity (%)(A_red_-A_ox)_/ A_red_)x100
**Glucose**	0.21±0.3	<1	0.02±0.4	91
**Maltose**	24.0±0.7	20	4.4±0.1	82
**Maltotriose**	125.6±0.6	100	27.7±0.5	78
**Maltotetraose**	129.1±2.5	103	28.8±0.1	78
**Maltopentaose**	145.3±2.8	116	22.2±0.2	85
**Maltohexaose**	112.7±2.5	90	16.7±0.1	85
**Maltoheptaose**	100.0±8.2	80	13.1±0.4	87
**Maltooctaose**	99.3±1.9	79	13.5±0.1	86
**Glycogen**	235.2±19.7	187	9.8±0.2	96
Glycogen*	4,276*	3,404*	178*	96*

*normalized to the non-reducing end (NRE) molarity in MOS. MOS NRE molarity (equivalent to MOS molarity): 10^−2^ mol L^-1^; glycogen NRE molarity: 5.5x10^-4^ mol L^-1^; ADP-glucose molarity: 10^−3^ mol L^-1^; *At*SS1 molarity: 7.6x10^-8^ mol L^-1^. Glycogen branching density was 9% and the total number of glucose residues is 5.5x10^4^ [28].

### *At*SS1 has a distinct redox response *in vitro*

**Fig 1:**
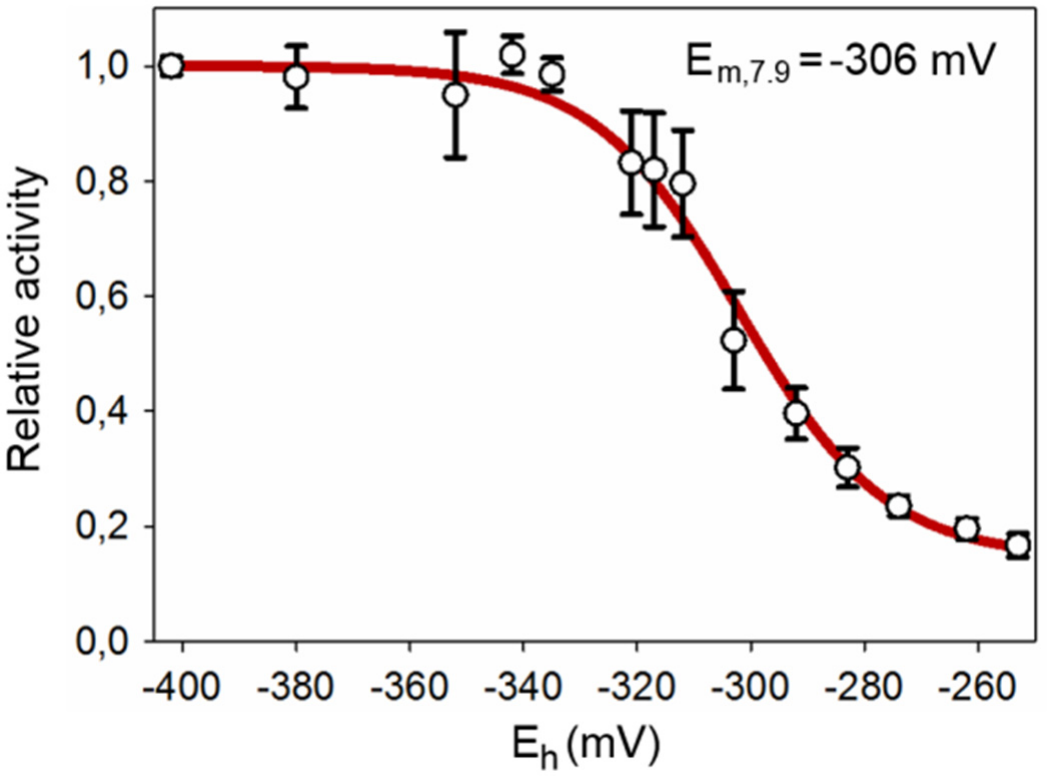
Redox titration of *At*SS1. The effect of the redox potential on *At*SS1 activity at pH 7.9 was determined by incubation in defined DTTred:DTTox ratios and subsequent activity assays. The activity was assayed by SCGA using maltotriose as acceptor. The titration data was fitted by non-linear regression using the Nernst equation anticipating a two-electron transfer process for the thiol-disulfide exchange reaction to calculate midpoint potentials. Values are the mean of two independent experiments (±SD).

In order to clarify whether the *At*SS1 activity response falls within a physiologically relevant redox potential range, we conducted a redox titration analysis. The activity of the protein was at the maximum level in the redox potential range between -410 mV and -350 mV, (Fig 1). A clear activity drop was observed at potentials around -340 mV to -300 mV. The midpoint potential for the *At*SS1 wild type was calculated to -306 mV. Based on these results, *At*SS1 wild type protein is predicted to be reduced and active during the light period and active photosynthesis.

### Trxs and NTRC are efficient catalysts for reactivation and deactivation of *At*SS1

**Fig 2:**
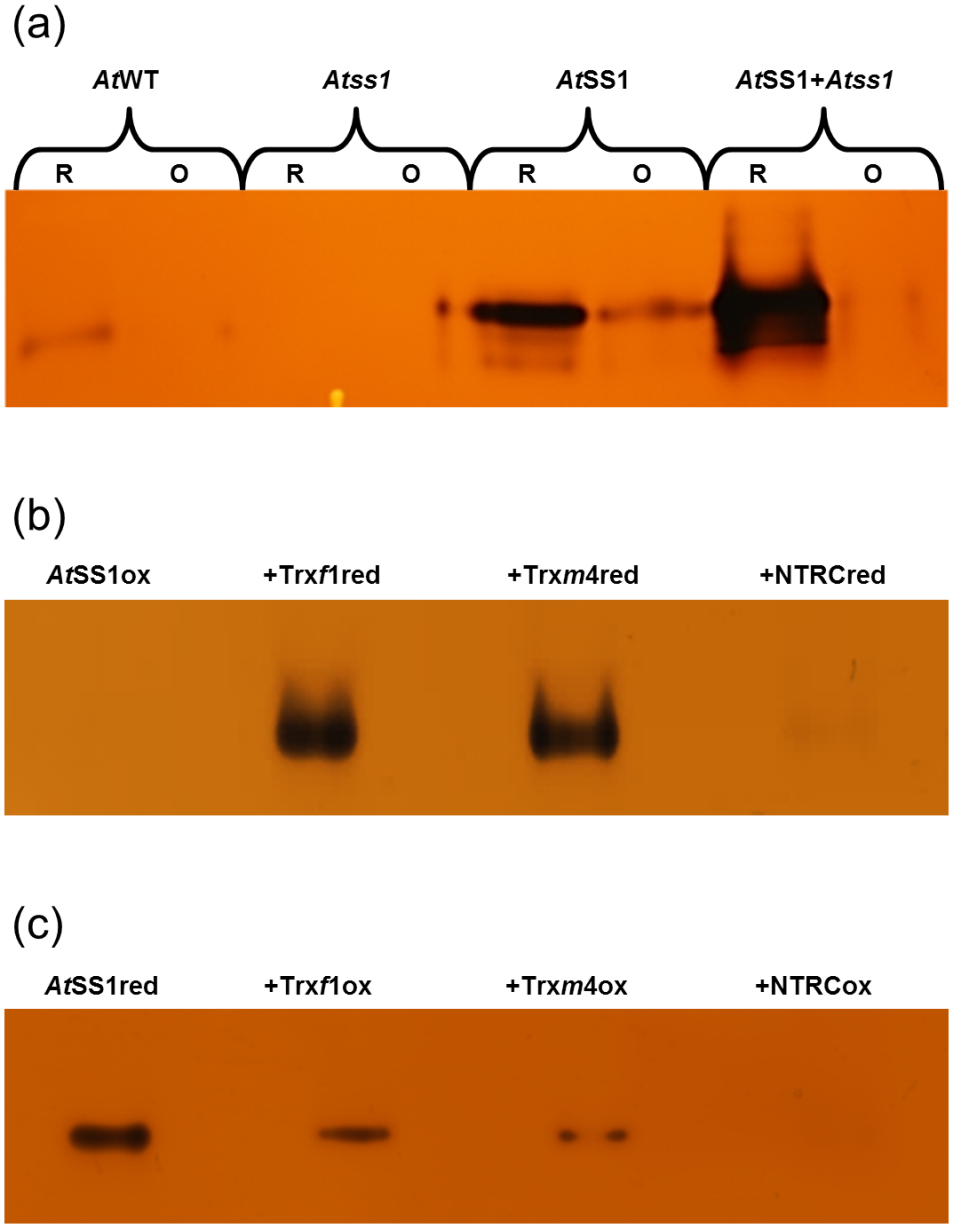
Reactivation and deactivation of recombinant *At*SS1 by Trx and NTRC. Isoform specific enzyme activity was monitored using glycogen-containing NAG. (a) Activity of *At*SS1 from Arabidopsis leaf extract (25 μL of 200 mg mL^-1^ extract) or recombinant protein (1 μg) combined with leaf extract from *Atss1*. (b) Oxidized *At*SS1 (2 μM) incubated with water, 10 μM reduced Trx*f*1, 10 μM reduced Trx*m*4 or 10 μM reduced NTRC as indicated. Trxs and NTRC (100 μM) were reduced with 0.5 mM DTTred. (c) Reduced *At*SS1 (2 μM) incubated with water, 10 μM oxidized Trx*f*1, 10 μM oxidized Trx*m*4, 10 μM oxidized NTRC as indicated. Trxs and NTRC (150 μM) were oxidized with 300 μM CuCl_2_.

To investigate whether leaf extract from Arabidopsis knock-out plants lacking SS1 activity (*Atss1*) can facilitate redox dependent activity modulation of recombinant *At*SS1, the recombinant *At*SS1 was treated with 20 mM DTTred or 20 mM oxidized dithiothreitol (DTTox) in the presence or absence of *Atss1* leaf extract. Enhanced activation and deactivation were observed when recombinant *At*SS1 was co-incubated with *Atss1* leaf extract (Fig 2a). These data suggest that physiological catalysts that can mediate this effect are present in the *Atss1* extract. To test this hypothesis recombinant Arabidopsis thioredoxin *f*1 (Trx*f*1), thioredoxin *m*4 (Trx*m*4) and NADPH-dependent thioredoxin reductase C (NTRC) were produced. DTT mediated pre-oxidized or pre-reduced *At*SS1 was incubated with DTT mediated pre-reduced or pre-oxidized Trxs/NTRC, respectively. Subsequently, enzyme activity was analyzed by either glycogen-containing NAG (Fig 2b and 2c) or by SCGA (Table 2).

**Table 2:** *At*SS1 activation and deactivation mediated by recombinant *At*Trxs. Reactivation of oxidized *At*SS1: protein was first oxidized by CuCl_2_, and then it was desalted and treated with water, reduced Trxs and DTTred. Trxs (100 μM) were reduced with 0.5 mM DTTred. Deactivation of reduced *At*SS1: protein was first reduced by DTTred, and then it was desalted and treated with water, oxidized Trxs and CuCl_2_. Trxs (150 μM) were oxidized with 300 μM CuCl_2_. Fully reduced sample was set as 100%. Desalting control (sample treated identically to oxidized Trxs but Trxs were omitted) was used to test that CuCl_2_ left after desalting is not the major deactivating factor. Activity was assayed by SCGA using maltotriose as acceptor; results are the mean of two technical replicates (±SD).

Reactivation of oxidized *At*SS1			Deactivation of reduced *At*SS1		
Treatment	Activity		Treatment	Activity	
	Absolute	Relative to maximum (%)		Absolute	Relative to maximum (%)
**water**	1.7±0.1	1.2±0.1	**water**	189.5±2.9	100.0±0.4
**10 μM DTTred**	5.4±0.7	3.8±0.5	**desalting control**	84.6±1.7	71.9±0.5
**10 μM reduced Trx*f*1**	35.3±1.7	22.9±1.1	**10 μM oxidized Trx*f*1**	2.0±0.2	0.8±0.0
**10 μM reduced Trx*m*4**	24.3±2.3	14.8±1.4	**10 μM oxidized Trx*m*4**	2.7±0.3	0.5±0.1
**20 mM DTTred**	140.4±7.1	100.0±5.0	**10 μM CuCl**_**2**_	2.3	1.1±0.2

Both approaches illustrate the ability of Trxs and NTRC to activate and deactivate *At*SS1 *in vitro*. As deduced from the NAG results, the activation degree was highest for *At*Trx*f*1 and decreased in the order *At*Trx*f*1, *At*Trx*m*4, *At*NTRC, while the deactivation degree increased in the same order. These data suggest that Trx*f*1 is the most efficient reductive enzyme activator, while NTRC is the most efficient oxidative enzyme deactivator.

### Modeling supports a regulatory disulfide between the N-terminal and C-terminal domains

**Fig 3:**
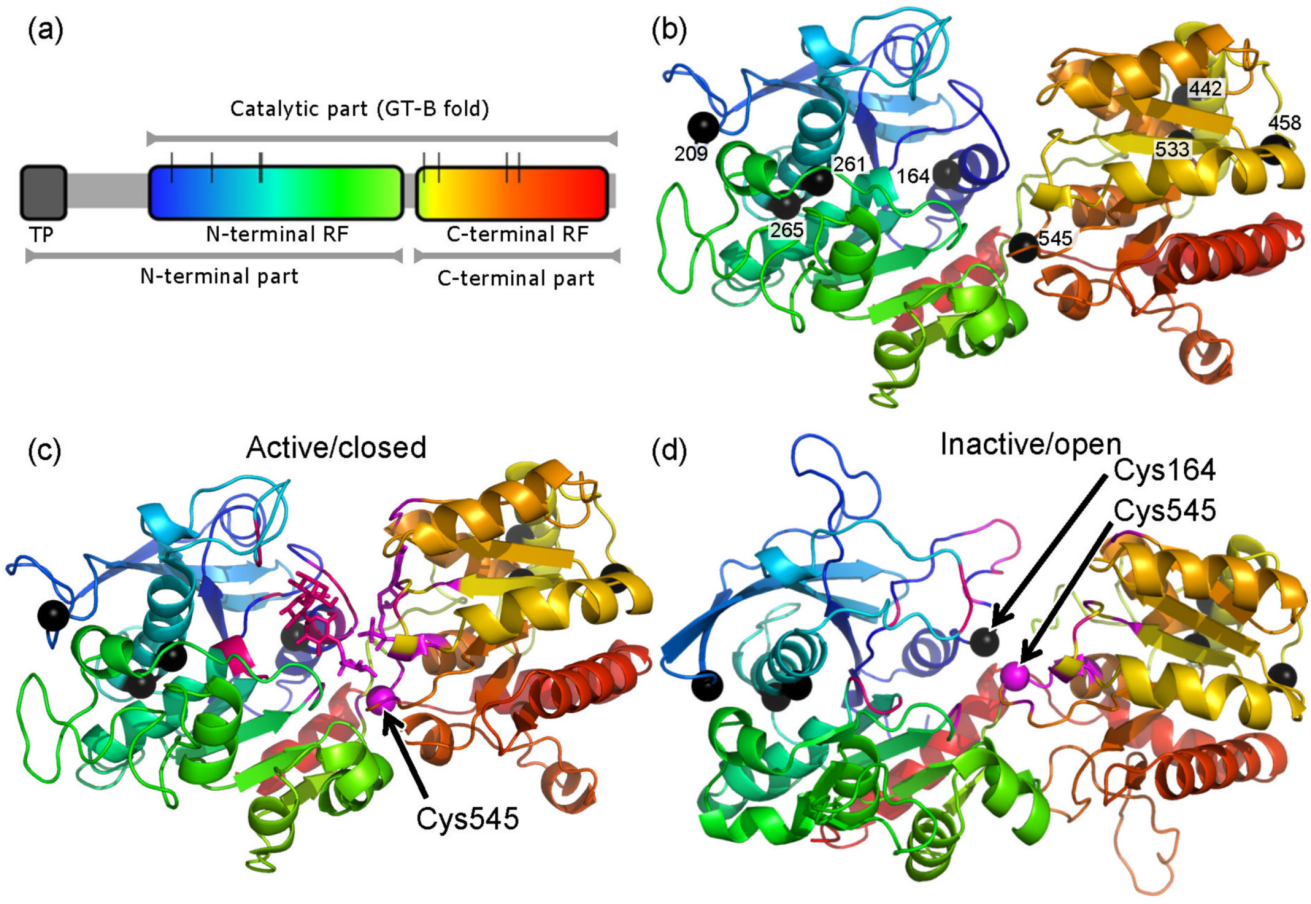
Structure models of *At*SS1 in closed and open conformations. (a) Domain organization showing the transit peptide (TP) and the eight Cys residues (black vertical bars). The two RFs are rainbow gradient color-coded showing the N-terminal RF in blue-green shades and the C-terminal in yellow-red shades. (b) Ribbon representation of an *At*SS1 modeled based on the *Ec*GS 2qzs template [32] color-coded as in (a). (c) The catalytically active closed conformation based on the *Ec*GS template showing the positions of ADP-glucose and maltotriose in the active site. ADP-glucose and its interacting amino acid residues (magenta) maltotriose with its interactions (pink), Cys residues (black spheres) except Cys545 (pink). (d) The catalytically inactive open conformation based on the *Hv*SS1 4hln template [30]. ADP-glucose and its interacting amino acid residues are indicated in magenta and maltotriose with its interactions are indicated in pink. Cys residues are shown as black spheres.

The proximity of Cys residues and likelihood of disulfide bridge formation and possible conformational strains in *At*SS1 was assessed by structure modeling using the SWISS-MODEL server [31]. The full-length *At*SS1 is a 72.1 kDa protein. The first 49 amino acid residues are predicted to be the chloroplastic transit peptide and are followed by a presumably unstructured region which in turn is followed by a largely N-terminal Rossmann fold domain which includes a helix at the C-terminus of the protein (residues 133–409 and 631–652), a small linker and a second Rossmann fold domain (residues 419–630) here termed the C-terminal, illustrating its overall position in the sequence (Fig 3a and 3b). The mature *At*SS1 has eight Cys residues: Cys164, Cys209, Cys261, Cys265, Cys442, Cys458, Cys533 and Cys545. The first four Cys residues are found in the N-terminal and the last four in the C-terminal domains.

SS1, as deduced from X-ray structures of *E*. *coli* GS and *Hv*SS1, can adopt a closed and an open conformation (32,30). The cleft between the two domains forms the catalytic site in the closed conformation. The closed conformation of *At*SS1 was modeled with 2qzs [32] as a template (Fig 3c), which is the only structure available of a glycogen or starch synthase with acceptor and donor analogues bound, and supplemented with the maltotriose acceptor of the almost identical *Ec*GS structure 3cx4 [29]. *Ec*GS shares 33% identity with *At*SS1 over the catalytic domains but all important details of the model are also supported by modeling based on *Os*GBSS as a template (41% identity), although the available *Os*GBSS structures [33] are of lower resolution and detail. The open conformation of *At*SS1 was modeled with 4hln [30] as a template (Fig 3d). This structure is of a plant (barley) SS1 and shares 72% identity with *At*SS1. As this structure is in the open conformation, it lacks a functional active site but its high sequence identity to *At*SS1 confirms all relevant details that depend on the internal structure of each domain, which are similar to those in the model based on *Ec*GS.

The model suggests that Cys545 is important for both catalysis and redox regulatory disulfide formation. Cys545 is directly involved in binding the glucose moiety of ADP-glucose [32] and a GS mutant in this residue shows reduced activity [34]. Our model of the closed conformation places Cys545 in the immediate vicinity of the glucose to be transferred (Fig 3c). The indication that disulfide bridge formation in *At*SS1 is important for redox regulation comes from the open conformation crystal structure of barley SS1 (*Hv*SS1), which contains a disulfide bridge between Cys126 and Cys506, equivalent to Cys164 and Cys545 in *At*SS1 [30]. Our models show that formation of this disulfide is equally feasible in *At*SS1 with a similar conformation that would maintain both Rossmann fold domains apart from each other and interfere with formation of a competent active site. The minor but significant decrease in redox sensitivity of a C506S mutant in *Hv*SS1 (70%, calculated based on data from [30]) suggests the presence of a more complex mechanism of redox regulation. The involvement of other cysteines in redox-mediated activity changes cannot be excluded. Moreover, the involvement of additional mechanisms of redox regulation acting in parallel cannot be excluded. As an example, we found NADP^+^ to inhibit *At*SS1 activity *in vitro* (S2 Fig and suppl. text). This effect could be due to either direct binding of NADP^+^ or oxidation of *At*SS1 causing disulfide formation. More investigations are needed to clarify whether such inhibition occurs at physiological NADP^+^ concentrations, which are reported to be 0.4 mM in the dark [35].

### All Cys residues affect both activity and redox sensitivity

**Fig 4:**
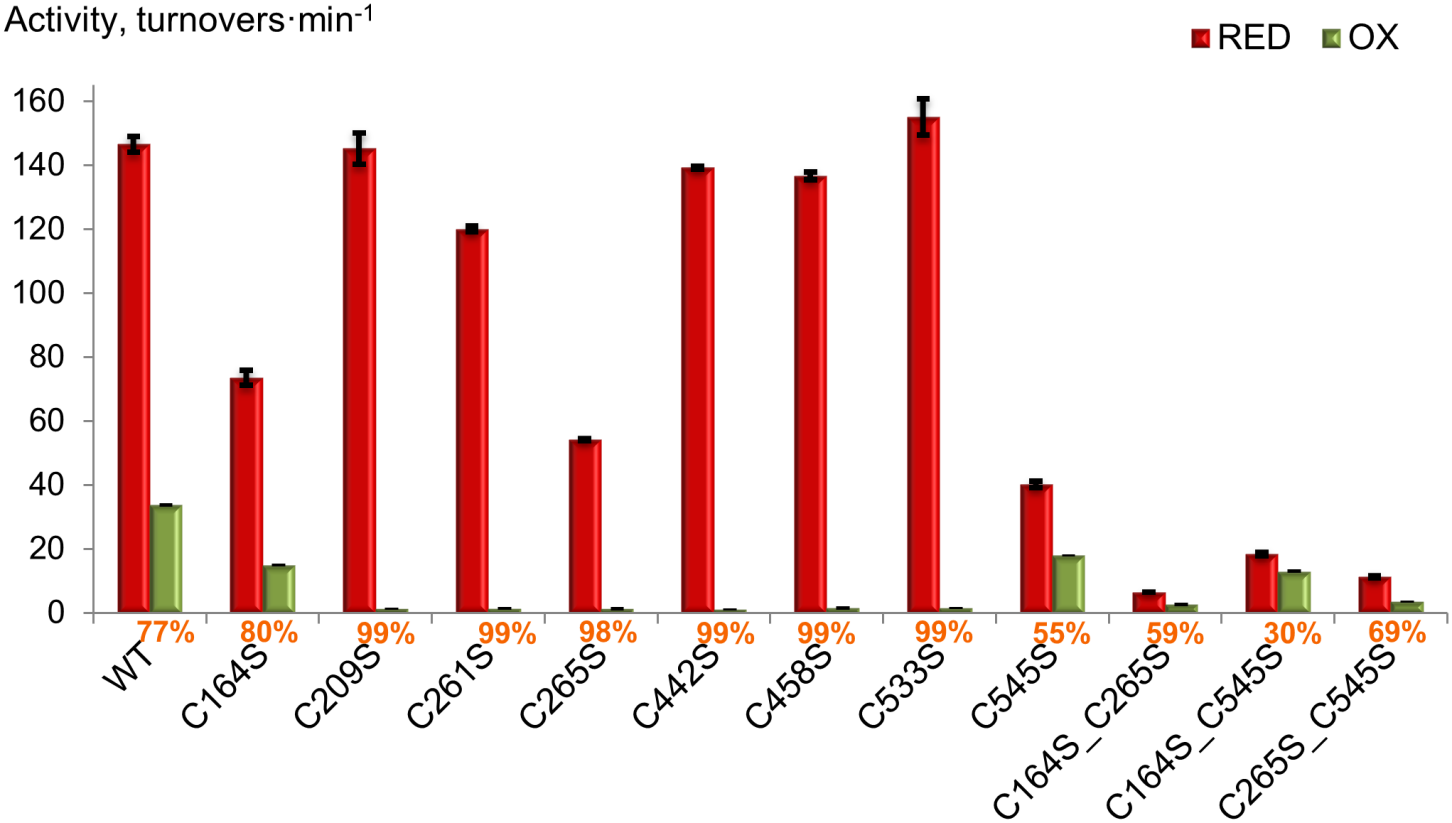
Activity and redox sensitivity of *At*SS1 wild type and mutant proteins. Protein activities were analyzed by SCGA using maltotriose as acceptor after treatment with either 20 mM DTTred (red bars) or 20 mM DTTox (green bars). Redox sensitivity is represented as percentage and values are given below the bars. Activity is expressed as enzyme turnovers min^-1^. The values represent the mean of two technical replicates (±SD).

A site-directed mutagenesis approach was employed to identify the Cys residues involved in the redox mediated modulation of *At*SS1. Initially the wild type protein and eight single cysteine-to-serine mutants were examined for catalysis and redox sensitivity. Based on results from an initial screening test, double mutants were produced and tested. The activity of each protein type in both reduced and oxidized form was measured by incubation with either 20 mM DTTred or 20 mM DTTox (Fig 4; S2 Table).

Impaired activity was found for the reduced C164S, C265S and C545S single mutants and for all the double mutants. The 50% lowered activity of C164S as compared to wild type *At*SS1 can be explained by the fact that Cys164 is only two amino acid residues away from the active site KXGGL motif (S3 Table). The decreased activity of C545S (27% of wild type activity) was expected since this Cys residue is directly involved in ADP-glucose binding [34]. Decreased activity of C164S_C545S (13%, S2 Table) and C265S_C545S (8%, S2 Table), as compared to wild type, could be explained by a combinatorial effect of the above mentioned single mutations.

The lower activity of C265S (37%) was unexpected since this cysteine is not in close proximity to the catalytic cleft. Potentially, C265 plays an important role in the correct folding of the N-terminal RF in the chloroplast. Alternatively, Cys164 and Cys265 might be required for formation of semi-folded intermediate(s) to stabilize *At*SS1 protein translated in the cytosol prior to transport into the plastid for the final folding. These notions are based on the low protein expression level in *E*. *coli* (S3 Fig) and the reduced activity of the C164S_C265S double mutant (4%, S2 Table) but needs verification.

Redox sensitivity was affected in every mutant except for C164S (Fig 4). The lowest redox sensitivity among the single mutants was for C545S (55%) and the overall lowest redox sensitivity was for the double mutant C164S_C545S (30%). This is consistent with our prediction derived from homology modeling of *At*SS1 based on the oxidized and inactive *Hv*SS1 crystal structure. Thus, a disulfide bridge formed between Cys164 and Cys545 could be the main structural basis of the oxidative inactivation. The unchanged level of RS of the C164S mutant could be explained by the possible interaction between the redox sensitive Cys545 and an alternative cysteine. The nearly complete redox sensitivity (98–99%) of all the remaining single mutants (C209S, C261S, C265S, C442S, C458S and C533S) indicates that the replacement of these cysteines by serine leads to an *At*SS1 protein structure, which is more susceptible to oxidation.

### Cys545 is both catalytically active and redox sensitive

To determine if Cys residues other than Cys545 influence ADP-glucose binding in *At*SS1, a Michaelis-Menten donor kinetics analysis was carried out. *At*SS1 mutants were treated with 20 mM DTTred or DTTox and activity was analyzed by SCGA with maltotriose as acceptor (S4 Table). The V_max_ values reflected similar tendencies as the redox screening data (Fig 4). The three single mutants C164S, C265S, C545S and all the double mutants showed decreased levels of activity as compared to the wild type protein. Redox sensitivity calculated using V_max_ values also confirmed the previous data.

The K_m_ value towards ADP-glucose for the reduced *At*SS1 wild type protein was 0.076 mM, which is in good agreement with previous data for recombinant *At*SS1 using glycogen as an acceptor (0.075 mM, [28]). The K_m_ values were significantly changed for the mutants C545S (9.7-fold increase), C164S_C545S (16-fold increase) and C265S_C545S (11-fold increase). The C261S and C164S_C265S mutants showed a moderate increase in K_m_ values (2.44- and 4.22-fold increase, respectively). Thus Cys545 was verified to be crucial for ADP-glucose binding. The lower activity of the C164S and C265S mutants could not be explained by low ADP-glucose binding indicating that these Cys residues are important for maintaining other structures in *At*SS1. Selected mutants were also analyzed by redox titration supporting the redox screening and Michaelis-Menten kinetic analyses (S4 Fig; S5 Table).

### Cysteines support protein folding and stability

The observed general decrease in *At*SS1 activity together with the almost complete redox sensitivity (98–99%) for all the single mutants except for C164S and C545S (Fig 4; S4 Table) suggested a possible involvement of these Cys residues in enzyme stability upon oxidation. To test this idea we performed a reactivation test (see details in the Table 3 caption) similar to the reversibility test (S1 Fig). Reactivation degree was calculated as the percentage of recovered activity relative to the activity of the fully reduced enzyme. All mutants displayed a decreased degree of reactivation compared to the wild type enzyme even though the extent of decrease differed among the mutants (Table 3). For cysteines located in the N-terminal RF (Cys209 and Cys261) the difference was not as pronounced. Cys residues in the C-terminal RF, however, were more important for protein stability showing a 44%, 35% and 9.5% reactivation for the C533S, C458S and C442S, respectively. Thus Cys442 is crucial for the stability of the enzyme upon oxidation.

**Table 3:** Reactivation of wild type *At*SS1 and mutant variants after oxidative inactivation. Recombinant *At*SS1 was either untreated or treated with 0.3 mM dithiothreitol (DTTred) or 5 μM CuCl_2_. Subsequently, proteins were desalted to remove reducing or oxidizing agents and the reduced (DTTred) protein was incubated with water (2. reduced), while the oxidized (CuCl_2_) protein was incubated with either water (3. oxidized) or 0.3 mM DTTred (4. reactivated). Enzyme activity was analyzed by SCGA with maltotriose as acceptor. The lower activity for reduced protein samples, as compared to values reported in Table 1, S2 and S4 Tables and Fig 4, is due to the different purification method applied (see details in the Supporting Information, S1 Protocol).

Protein	Absolute activity, turnovers min^-1^				Relative activity
	1. Untreated	2. Reduced	3. Oxidized	4. Reactivated	5. Reactivation (%)
**WT**	53	92.7	1.70	78.6	85
**C209S**	1.52	80.2	1.44	53.2	66
**C261S**	1.88	79.4	1.40	55.7	70
**C442S**	1.45	90.2	1.18	8.53	9.5
**C458S**	1.45	73.5	0.77	26.0	35
**C533S**	1.13	78.8	1.21	34.9	44
**C164S_C545S**	7.06	10.4	3.66	5.09	49

We speculate that the observed effects could be explained by the fact that the formation of the inter-domain bridge depends on the geometry of the N- and C-terminal folds. Possibly the *At*SS1 wild type protein folds in a manner separating key Cys residues from each other, thereby preventing oxidative bridging, especially in the absence of Trxs. Replacement of every single Cys residue by serine may cause a disruption of normal protein folding and result in a protein with the key Cys residues being relatively close to each other and thus being more susceptible to oxidation even for the untreated sample.

### The cysteine content correlates positively with organism complexity

The apparent importance of Cys residues for *At*SS1 catalysis and stability prompted us to look into the evolutionary origin of these amino acid residues. Initially SS1 homologs were identified among *Embryophyta*, *Charophyta*, *Chlorophyta*, *Bacteria* (including *Cyanobacteria*) and *Archaea*. A multiple alignment of these sequences were analyzed for the presence or absence of Cys residues (Table 4). Interestingly, the number of Cys residues equivalent to the *At*SS1 Cys residues correlated positively with organism complexity. The Cys residue distribution within the *Embryophyta* was essentially similar to that of *At*SS1 with the exception of the Cys261 equivalent, which is missing in some dicots, and the Cys458 equivalent missing in monocots, *Lycopodiophyta* and *Bryophyta*. The cysteine residue equivalent to Cys545 in *At*SS1 is the most conserved Cys residue, being present in nearly all species (except for *Thermococcus onnurineus* and *Manihot esculenta*). At the same time, the Cys545 equivalent is the only Cys residue present in *Archaea* and *Bacteria* (except for *Methanococcus maripaludis*). The relative content of Cys residues was higher for the more complex species, with an average of 1.7% for monocots and dicots and 0.9% for *Bacteria* (S5 Fig).

**Table 4:** Summary of *At*SS1 equivalent Cys residues in aligned sequences. The presence of an equivalent Cys residue is indicated with “+” and the absence is indicated with the corresponding amino acid substitution. Additional information including species taxonomy and protein sequence accession number is listed in the S6 Table.

Protein	Cys164	Cys209	Cys261	Cys265	Cys442	Cys458	Cys533	Cys545
***Dicotyledoneae***								
***Vv_SS1***	+	+	+	+	+	+	+	+
***Mt_SS1***	+	+	SER	+	+	+	+	+
***St_SS1***	+	+	SER	+	+	+	+	+
***Ca_SS1***	+	+	SER	+	+	+	+	+
***Gm_SS1***	+	+	+	+	+	+	+	+
***Me_SS1***	+	+	+	+	+	+	+	ARG
***Fv_SS1***	+	+	ALA	+	+	+	+	+
***Tc_SS1***	+	+	+	+	+	+	+	+
***Pt_SS1***	+	+	+	+	+	+	ALA	+
***Cs_SS1***	+	+	+	+	+	+	+	+
***Monocotyledoneae***								
***Ta_SS1***	+	+	+	+	+	VAL	+	+
***Bd_SS1***	+	+	+	+	+	ILE	+	+
***Os_SS1***	+	+	+	+	+	VAL	+	+
***Zm_SS1***	+	+	+	+	+	VAL	+	+
***Hv_SS1***	+	+	+	+	+	VAL	+	+
***Lycopodiophyta***								
***Sm_SS1***	+	+	+	+	+	VAL	+	+
***Bryophyta***								
***Pp_SS1***	+	+	+	+	+	VAL	+	+
***Charophyta***								
Kf_SS1	ILE	+	+	+	SER	VAL	+	+
***Chlorophyta***								
***Ot_SS1***	+	+	ALA	+	+	VAL	+	+
***M_sp_SS1***	+	+	SER	+	ASN	VAL	+	+
***Mp_SS1***	+	+	SER	+	+	VAL	+	+
***Bp_SS1***	+	+	ALA	+	+	VAL	VAL	+
***Cr_SS1***	LEU	PHE	ALA	-	+	ILE	ALA	+
***Vc_SS1***	MET	GLY	+	+	+	ALA	SER	+
***Cyanobacteria***								
***N_sp_GS***	VAL	LYS	SER	ALA	ASN	ALA	THR	+
***C_sp_GS***	VAL	GLN	ALA	ALA	ASN	THR	ILE	+
***S_sp_GS***	VAL	LYS	SER	ALA	ASN	ALA	ALA	+
***Pm_GS***	VAL	THR	ALA	ALA	ASN	LYS	SER	+
***Gv_GS***	VAL	THR	SER	ALA	ASN	ARG	ALA	+
***Bacteria***								
***Ec_GS***	ILE	THR	GLY	ALA	ASN	VAL	ALA	+
***Ta_GS***	VAL	TYR	SER	PHE	ASN	VAL	SER	+
***Euryarchaeota***								
***To_GS***	VAL	ALA	PHE	SER	ARG	GLY	VAL	PHE
***Mf_GS***	MET	TYR	ALA	VAL	SER	THR	SER	+
***Mm_GS***	LEU	TYR	ALA	VAL	SER	THR	+	+

The Cys164 equivalent appears for the first time among the *Chlorophyta* and *Ostreococcus tauri*, *Micromonas sp*. and *Bathycoccus prasinos* (all belonging to the *Mamiellophyceae*) possess this cysteine residue while *Chlamydomonas reinhardtii* and *Volvox sp*. (*Chlamydomonales*) do not. Remarkably Cys265 and Cys442 which were found to be important for *At*SS1 folding and stability, respectively, appear within the same taxon alongside the Cys164 equivalent. The relative cysteine content was also higher in species where the Cys164 equivalent is present. At the same time, the SS1 protein from *Klebsormidium flaccidum* (*Charophyta*), which is the ancestral taxon for modern plants [36], does not have this Cys residue equivalent.

The presence of the Cys164 equivalent, the number of Cys residues equivalent to the Arabidopsis Cys residues and the relative content in the analyzed proteins, reveal a positive correlation with organism complexity. The fact that the cysteine content increases within the *Chlorophyta* suggests that these amino acid residues might have evolved as a response to compartmentalized and co-localized photosynthesis and starch biosynthesis. The lack of a Cys164 equivalent in *Charophyta* could imply that cysteine-mediated redox regulation evolved multiple times.

## Discussion

### The redox-active Cys residues of *At*SS1 are important for catalysis

The recombinant *At*SS1 analyzed in this study demonstrates a redox sensitivity similar to that of native *At*SS1 [25]. *At*SS1 was found to be redox sensitive regardless of the glucan acceptor types tested, while redox sensitivity was higher with glycogen as a substrate than MOS. This might be explained by simultaneous binding of glycogen to the N-terminal and C-terminal RF of *At*SS1 maintaining a more open conformation of the enzyme, thus making redox-active Cys residues more accessible for oxidation catalysts. The ability of *At*SS1 to be activated and deactivated by the *in planta* redox transmitters (Trx*f*1, Trx*m*4 and NTRC) was confirmed with Trx*f*1 as the most effective activator. This is supported by previous investigations showing that Trx*f*1 is the most efficient physiological reductant for many chloroplastic enzymes, including the starch metabolic enzymes *At*AGPase [16], *St*AGPase [37], *St*GWD [24], *At*SEX4 [38], *At*BAM1 [39,40], and *At*AMY3 [41].

The increased efficiency of NTRC over Trxs in redox deactivation, as demonstrated in this study, could indicate a hitherto unexplored role of NTRC as a redox inactivator of chloroplast enzymes. It is interesting to note that using a NADPH-dependent thioredoxin reductase as a redox inactivator might permit recovery of reducing equivalents as NADPH. The same may not be feasible using the FTR system due to the much more negative redox potential of Fd. Further experiments may shed more light on this little-studied aspect of thioredoxin redox regulation.

Combining experimental evidence and homology modeling using barley SS1 as a template we identified two redox-active Cys residues, Cys164 and Cys545, supporting our hypothesis that reversible disulfide bridge formation upon oxidation is the main structural mechanism of protein inactivation. Both cysteines are suggested to play important roles in enzyme catalysis, Cys545 in ADP-glucose binding and Cys164 in acceptor binding, although the latter needs to be verified for *At*SS1. Previous studies on the glucan phosphatase SEX4 [38] and the α-amylase AMY3 [41] suggest that catalytic Cys residues are involved in post-translational redox regulation in starch metabolism. In these cases reductive activation not only induces a conformational change permitting full enzymatic activity, but also provides direct access to catalytically active amino acid residues.

### The low reducing midpoint potential indicates protein stabilization at highly oxidative conditions

The midpoint potential for the *At*SS1 is the least negative midpoint potential (-306 mV) reported so far for starch metabolizing enzymes: -350 mV for *At*BAM1 [39], -329 mV for *At*AMY3 [41], -310 mV for *St*GWD [24], all determined at pH 7.9. These results as well as midpoint potentials documented for Calvin-Benson cycle enzymes [42,43] together with the assertion that the overall redox potentials of the chloroplast stroma approach those of the cytosol in the dark [44], could indicate that this enzyme retains its active (reduced) conformation in the dark and does not encompass an absolute light-dark activity switch. More likely, its redox-active disulfides could serve in a protective mechanism for catalytic Cys residues under severe oxidative stress conditions which are often the consequence of various types of biotic and abiotic stress [45]. Oxidative conformational change not only masks catalytic amino acid residues but also directly inactivates them by disulfide formation. In this way reversible inactivation provides a protective mechanism preventing irreversible modification of important Cys residues by *e*.*g*. sulfonation. Since the chloroplast is the main source of reducing equivalents and reactive oxygen species (ROS), the existence of such a post-translational mechanism can combine sensing of the photosynthetic rate with antioxidant protection, both of which are important for sustained dynamics in starch biosynthesis in response to various environmental cues.

### Cys residues play multiple roles in *At*SS1 function

The experimental data indicate multiple physiological roles of Cys residues including maintenance, protection and fine regulation of *At*SS1 activity. The decreased activity of the C545S, C164S and C265S mutants demonstrated their importance for catalysis, but the exact mechanism by which the latter two Cys residues influence activity remains to be explored. Dynamic cysteine disulfide exchange takes part in e.g. post-translational protein folding and stabilization [46] and Cys265 together with Cys164 could be crucial for proper protein folding in the plastid [47] and/or in forming intermediate folding structures in the cytosol. As deduced from the decreased reactivation level after oxidation, Cys442 plays an important role in enzyme stability upon oxidation. Other Cys residues, including Cys209, Cys261, Cys458 and Cys533, were also important for enzyme function (Table 5), but the exact mechanism could not be established by the experimental approach used in this study.

**Table 5:** Summary of the suggested roles of individual Cys residues in *At*SS1. N.D. not determined.

Cys residues	Observed effects for proteins					Possible role of the Cys residue(s)
	Reactivation (%)	V_max_	K_m_, fold change	Redox sensitivity (%)	Protein production	
**WT**	85	100	1.00	77	Normal	N.D.
**C164S**	N.D.	36	1.33	80	N.D.	Catalysis (possibly in acceptor binding). Redox regulatory disulfide with Cys545. Protein folding (intermediate disulfide with Cys265)
**C209S**	66	90	1.67	99	N.D.	Protein folding. Alternative partner for Cys545, Cys164
**C261S**	70	95	2.44	99	N.D.	Protein folding
**C265S**	N.D.	13	0.96	98	N.D.	Protein folding. Catalysis (rather indirect influence)
**C442S**	9.5	86	1.28	99	N.D.	Protein folding. Critical for stability upon oxidation
**C458S**	35	88	1.32	99	N.D.	Protein folding. Important for stability upon oxidation
**C533S**	44	93	1.41	99	N.D.	Protein folding. Important for stability upon oxidation
**C545S**	N.D.	38	9.7	55	N.D.	Catalysis (binds glucose to be transferred to the acceptor). Redox regulatory disulfide with Cys164
**C164S_C265S**	N.D.	5	4.22	59	Very low yield. Truncated protein	Critical for protein folding
**C164S_C545S**	49	28	16.0	30	Low yield	Main redox regulatory disulfide
**C265S_C545S**	N.D.	9	11.0	69	Very low yield	Catalysis (combinatorial effect of the two mutations)

The higher cysteine content found in eukaryotic SS1 proteins as compared to prokaryotic GS illustrates a directional selection for higher cysteine number. These data are in a good agreement with a previous phylogenetic complexity study. By using hundreds of proteins from various species a positive correlation was found between cysteine occurrence and organism complexity [48].

The fact that both absolute and relative cysteine content of *At*SS1 protein starts to increase within the *Chlorophyta* indicates a function of these amino acid residues in chloroplast localized starch biosynthesis. Our experimental data reveal importance of at least two Cys residues which are involved in a reversible thiol-disulfide exchange. If the presence of these residues is indicative of redox regulation, this mechanism appears to predate the evolution of land plants and may have evolved with the photosynthetic eukaryotes. This idea is supported by a very recent evolutionary analysis of redox regulation in chloroplasts concluding that thiol-based redox mechanisms are more pronounced in eukaryotes than the prokaryotic cyanobacteria, and in land plants as compared to aquatic species, due to higher light intensities [49]. However, as discussed above, plastidial compartmentation of starch metabolism in higher organisms suggests involvement of additional Cys residues in protein folding inside the plastid and/or intermediate protein folding in the cytosol to stabilize translated protein.

### Redox regulated starch biosynthetic enzymes form an integral part of carbon allocation

Reductive activation of starch biosynthesis is intuitively more relevant as compared to reductive activation of starch degradation [50] since this mechanism links the production of reducing power by active photosynthesis to starch accumulation during the day. Similarly, the majority of enzymes in the Calvin-Benson cycle undergo diverse post-translational modifications including reversible disulfide bond formation catalyzed by Trxs. This mechanism could thus be a promising target for biotechnological improvement of starch yield using a complementing gene-overexpression strategy and a few studies supporting this idea have been conducted. First, complementation of an Arabidopsis KO mutant with redox-insensitive AGPase lead to increased levels of ADP-glucose, maltose and starch [23]. Second, Arabidopsis mutants lacking Trx*f*1 demonstrated decreased light-activation of AGPase and increased starch accumulation [16] and tobacco plants overexpressing Trx*f* showed up to a 700% increase in starch content although the level of redox-dependent AGPase activation was unaffected [15]. Third, Arabidopsis plants overexpressing NTRC accumulated increased levels of starch in illuminated leaves [51] and fourth, an increased level of trehalose-6-phosphate (Tre6P) was accompanied by an increase in the redox activation state of AGPase and enhanced starch synthesis in Arabidopsis leaves [52]. We propose that the above-mentioned effects can be explained by the coordinated reductive activation of several starch biosynthetic enzymes and not just AGPase. Such coordination also advocates for the presence of a shared carbon flux control mechanism among the majority of the starch biosynthetic enzyme, and could serve to maintain balanced activities of starch biosynthetic enzymes for correct structuring of the starch granule as previously suggested [25,53].

Alternatively, a redox dependent thiol-disulfide exchange mechanism could play a protective role to provide enhanced stability upon oxidative stress. For example, the enhanced affinity for the starch granule surface in the oxidized state demonstrated for GWD1 [24] and SS1 (S6 Fig), could be physiologically relevant if such protein immobilization upon oxidation decreases the risk of unwanted proteolysis. Additional mechanisms of thiol modification such as nitrosylation and glutathionylation, which are widely distributed among the enzymes of the Calvin-Benson cycle [42], have yet to be explored in the context of starch metabolism regulation.

The redox sensitivity of *At*SS1 demonstrated in this study could serve as a potential target for controlling the carbon flux to and from starch during the day and night, respectively. The structural basis of the post-translational redox modification determined for *At*SS1 could be extrapolated to other starch synthase isoforms due to high conservation of the Cys545 equivalent among all the SS enzymes. The apparent multiple roles of Cys residues highlight the potential of these amino acid residues for targeted stress-tolerant enzyme bioengineering for other transferases and enzymes in plant metabolism.

## Materials and Methods

### Structure modeling

The *At*SS1 protein structure was predicted by the SWISS-MODEL server (http://swissmodel.expasy.org; [31]) using two templates. The catalytically active conformation was modeled using *E*. *coli* glycogen synthase (*Ec*GS, PDB id 2qzs, 33% identity to *At*SS1) [32]. Coordinates for maltotriose bound to the active site were acquired from the structure of *Ec*GS with bound maltotriose (PDB id 3cx4, [29]) using the COOT software [54]. The catalytically inactive version was modeled using the *Hv*SS1 structure (PDB id 4hln, 72% identity) [30] as a template. All ribbon three-dimensional protein representations were produced in PyMol (www.pymol.org/).

### Cloning and mutagenesis of *At*SS1

The open reading frame of *Arabidopsis thaliana* starch synthase 1 (*At*SS1) (GenBank accession No: BAE98960.1) was synthesized as a codon-optimized construct for expression in *E*. *coli* by GenScript (http://www.genscript.com). The plastid target peptide (the first 49 aa) predicted by ChloroP 1.1 (http://www.cbs.dtu.dk/services/ChloroP/; [55]) was excluded. Additional NheI and XhoI restriction sites at the 5´ and 3´ ends, respectively, were included in the synthetic gene sequence and the construct was subsequently cloned into the pET28a expression vector (Novagen) using the Rapid DNA Ligation Kit (Fermentas). Using the pET28a-SS1vector as a DNA template eight single mutants were generated with the QuikChange site-directed mutagenesis protocol (Stratagene/Agilent Genomics). Each of the eight mutants carried a single cysteine to serine substitution: C164S, C209S, C261S, C265S, C442S, C458S, C533S, and C545S. The C164S_C265S and C164S_C545S double mutants were generated using the C164S mutant sequence as a template while the C265S_C545S double mutant was based on C545S. The PCR reaction product was cleaved with DpnI (New England BioLabs, R0176) and used directly for *E*. *coli* transformation (DH10β) with a standard protocol for transformation of chemically competent cells. All mutations were verified by sequencing.

### Production and purification of recombinant *At*SS1, *At*Trx*f*1, *At*Trx*m*4 and *At*NTRC

Proteins were produced as N-terminally 6xHis-tagged polypeptides in *E*.*coli* and purified using a HisTrap HP column (GE Healthcare) according to manufacturer instructions. Protein concentration was determined spectrophotometrically by Nanodrop 2000c (Thermo scientific). Extinction coefficients as well as all detailed protocol of recombinant protein production and purification, DNA, primer and protein sequences used in this study are given in S1 Protocol in Supporting Information.

### Default protocol for analysis of SS activity by spectrophotometric coupled glycosyltransferase assay (SCGA)

SS activity was determined essentially as previously described with a protocol [26] using ADP-glucose instead of UDP-galactose as a glucose donor. Enzyme assays were performed in a 100 μL volume containing 50 mM Bicine, pH 8.5, 25 mM potassium acetate, 2 mM MgCl_2_, 0.1% (w/v) bovine serum albumin (BSA), 0.375 mM NADH (Sigma, N8129), 0.7 mM phosphoenolpyruvate tricyclohexylammonium salt (Sigma, P7252), 6 U/ml pyruvate kinase, and 30 U/ml lactate dehydrogenase (both from Sigma, P0294) at 37°C. 10 mM maltotriose (Sigma, M8378) were used as an acceptor. *At*SS1 (90–200 nM) was added to a mixture containing the components mentioned above, incubated in a microplate at 37°C for 5 minutes with continuous mixing in the microplate reader and the reaction was initiated by addition of 1 mM ADP-glucose (Sigma, A0627 or enzymatically synthesized as described [30]). Enzymatic activity was monitored for 1 h as a decrease in NADH absorbance at 340 nm and the linear part of the reaction coordinate was used for activity calculation. Acceptor free or enzyme free samples were used as a blank.

### Redox reversibility of *At*SS1 activity

*At*SS1 protein (3.6 μM; 0.25 μg μL^-1^) was treated with 0.3 mM dithiothreitol (DTTred) or 5 μM CuCl_2_ and then desalted against 20 mM TRIS pH 8.0, 0.2 M NaCl, 10% (v/v) glycerol using PD SpinTrap G-25 columns (GE Healthcare) to remove reducing or oxidizing agents. For reversibility experiments, pre-reduced protein was treated with 5 μM CuCl_2_ and pre-oxidized protein was treated 0.3 mM or 20 mM DTTred. All treatments were done for 3.5 h at 37°C and enzymatic activity was assayed using the default protocol.

### Acceptor preference of *At*SS1

*At*SS1 protein (0.08 μg μL^-1^) was reduced with 20 mM DTTred or oxidized with 20 mM trans-4,5-dihydroxy-1,2-dithiane (DTTox) for 105 min at 37°C. Activity was assayed using the default protocol with the following acceptors; glucose (Merck, 1.08342.2500), maltose (Sigma, M5895), maltotriose (Sigma, M8378), maltotetraose (Sigma, M8253), maltopentaose (Sigma, M8128), maltohexaose (Sigma, M9153), maltoheptaose (Sigma, M7753), maltooctaose (Carbosynth, OM069411101), and glycogen from rabbit liver, type III (Sigma, G8876). All acceptors were used at a concentration of 10 mM except for glycogen which was 1 mg mL^-1^.

### Redox screening and titration

*At*SS1 and mutant variants (0.08 μg μL^-1^) were treated with 20 mM DTTred or 20 mM DTTox for 2 h at 37°C and enzymatic activity was assayed using the default protocol. Prior to redox titration, proteins were desalted over 100 mM TRICINE, pH 7.9. Redox equilibration was performed for 2 h at 37°C in 0.05 mL containing 100 mM TRICINE, pH 7.9 and 20 mM DTTred/DTTox in various thiol-disulfide ratios. The relationship between redox potential and DTTred/DTTox concentration ratio was calculated using the Nernst equation [56]. Final protein concentrations were 0.050–0.095 mg mL^-1^. Activity was assayed using the default protocol with 1 mM ADP-glucose for all proteins except for C164S_C545S (2 mM). The titration results were fitted by non-linear regression (SigmaPlot v.12.5) to the Nernst equation setting the value of *n* at 2 (anticipating a two-electron transfer process for the thiol-disulfide exchange reaction).

For Michaelis-Menten kinetic analysis enzyme activities were assayed using the default protocol with the following exceptions. SS1 proteins (6–14 μg) were treated for 2 h at 37°C with 20 mM DTTred or DTTox. For all proteins, except C545S and C164S_C545S, the following ADP-glucose concentrations were in the final assays; 0.01, 0.025, 0.075, 0.1, 0.15, 0.25, 0.5, 1 and 2 mM. For the C545S and C164S_C545S the ADP-glucose concentrations in the final assays were 0.05, 0.125, 0.25, 0.75, 1, 1.5, 2.5, 5 and 10 mM. ADP-glucose free samples were used as a control. V_max_ and K_m_ were calculated using the SigmaPlot software Enzyme Kinetic toolbox based on Michaelis-Menten kinetics.

### Reactivation and deactivation of *At*SS1 by *At*Trxs or *At*NTRC

For reactivation, recombinant *At*SS1 was oxidized with CuCl_2_ in a 1:2 molar ratio and recombinant Trxs and NTRC (100 μM) were treated with 0.5 mM DTTred and then desalted against 20 mM TRIS pH 8.0, 0.2 M NaCl, 10% (v/v) glycerol using PD SpinTrap G-25 columns (GE Healthcare) to remove reducing or oxidizing agents. The pre-oxidized SS1 protein (1–2 μM) was then incubated with either water, 10 μM DTTred, 20 mM DTTred, 10 μM pre-reduced Trx*f1*, 10 μM untreated Trx*f1*, 10 μM pre-reduced Trx*m4* or 10 μM untreated Trx*m4*. All treatments were for 1 h at 30°C. Reduction of Trxs was verified by the DTNB assay (see the *At*NTRC production protocol in S1 Protocol) and *At*SS1 activity was assayed using the default protocol.

For deactivation, *At*SS1 protein (12 μM) was pre-reduced with 0.5 mM DTTred and recombinant TRXs and NTRC (150 μM) were treated with 300 μM CuCl_2_. All samples were desalted three times as above. Pre-reduced SS1 protein (2 μM) was incubated with either water, 10 μM pre-oxidized Trx*f1*, 10 μM pre-oxidized Trx*m4*, 10 μM pre-oxidized NTRC or 10 μM CuCl_2_. All treatments were for 1 h at 37°C. SS activity was analyzed with the default protocol and with glycogen-containing native activity gel electrophoresis (NAG) as described below.

### Plant growth

*Arabidopsis thaliana* wild type Col-0 and *Atss1* mutants (FLAG_203C08) were grown from seed in potting compost in growth chambers at 20°C and 70% relative humidity with a 12/12 h photoperiod at a photon flux density of 120–150 μmol m^-2^ s^-1^. Leaves were harvested directly into liquid nitrogen 2 h before the end of the light period and kept at -80°C until analysis.

### Analysis of SS1 activity using native activity gel electrophoresis (NAG)

SSs were extracted from Arabidopsis leaf material in an ice cold buffer containing 100 mM BICINE pH 8.3, 1 mM EDTA, 10% (v/v) glycerol supplemented with protease inhibitor tablets (1 per 50 ml, Roche, 05056489001) using a ground-glass homogenizer (200 mg/ml of frozen powdered leaf material). Insoluble material was pelleted at 14 000 g for 10 min at 4°C and the supernatant was used immediately for NAG experiments. Electrophoresis gels were prepared as previously described [27] using either 0.8% (w/v) blue mussel glycogen (Sigma, G1508) or 0.15% (w/v) oyster glycogen (Sigma, G8751) as substrate in the separating gel. *At*SS1 protein (6–24 μM) was mixed with 5x loading buffer (0.05% bromophenol blue, 50% glycerol and 312,5 mM TRIS-HCl pH 6.8), loaded on the gel and run at 100–120 V for 3–3.5 h at 4°C. After electrophoresis the gels were washed twice in incubation buffer (100 mM BICINE pH 8.5, 0.5 M sodium citrate, 0.5 mM EDTA, 10% (v/v) glycerol) for 15 min and subsequently incubated in the same buffer supplemented with 1 mM ADP-glucose for 16 h at 37°C. Activities were revealed after staining with an iodine solution (0.34% I_2_, 0.68% KI (w/v)).

## Supporting Information

S1 Fig Reversible redox modulation of recombinant *At*SS1 activity. (a) Overview of the redox reversibility experiment. (b) Results obtained by the activity assays. The *At*SS1 protein was first treated with 0.3 mM dithiothreitol (DTTred) or 5 μM CuCl_2_ for reduction or oxidation of the enzyme, respectively. Following desalting, the pre-reduced protein was treated with 5 μM CuCl_2_ while the pre-oxidized protein was treated with DTTred (0.3 mM and 20 mM). The activity was assayed by SCGA using maltotriose as acceptor. The values represent the mean of two technical replicates (±SD). Red: reduced *At*SS1, Ox: oxidized *At*SS1. The activity of *At*SS1 pre-reduced with reduced dithiothreitol (DTTred) decreased to 1.8% when treated with CuCl_2_. The untreated protein (no DTT) oxidized with CuCl_2_ had activity close to zero (0.3%) but activity could be restored to 67% after treatment with 0.3 mM DTTred. The apparent loss of *At*SS1 activity following reactivation could be due to irreversible oxidation of redox-sensitive Cys residues.
(DOCX)

S2 Fig Influence of NADP^+^ on activity of *At*SS1. Enzyme activity was monitored by glycogen-containing NAG. (a) Activity of pre-reduced *At*SS1 protein treated with 0, 0.1, 0.5, 1.0 and 10 mM NADP^+^ or 10 μM of CuCl_2_. (b) Activity of pre-reduced *At*SS1 protein incubated with 2.5, 5, 7.5 and 10 mM NADP^+^, and incubated with either 1 mM ADP-glucose only (top gel) or 1 mM ADP-glucose supplemented with 20 mM DTTred (bottom gel). (c) Model of *At*SS1 showing the active site area. Blue: the N-terminal domain of *At*SS1, red: the C-terminal domain. Bound ADP-glucose (magenta) is represented by the ADP and glucose fragments unmodified from structure 2qzs [32] from the PDB. NADPH (green) has been modeled in a compatible conformation that avoids clashes with the protein. (d) Relative orientation of the nicotinamide moiety in the model (pink together with its ribose) and Cys545 (yellow). NADP^+^ was found to inhibit *At*SS1 activity *in vitro* (S2 Fig a). Analysis of the concentration dependence of NADP^+^ on *At*SS1 activity showed that the inhibition was reversible following incubation with 20 mM DTTred (S2 Fig b). To reveal a possible specific interaction between NADP^+^ and *At*SS1, a ThermoFluor assay of protein stability was performed. The rationale behind this experiment was that any specific interaction might stabilize the protein as detected by an increased melting temperature [57]. The results (S1 Table) demonstrate elevated thermostability of *At*SS1 protein pretreated with NADP^+^. This effect could be due to either direct binding of NADP^+^ or oxidation of *At*SS1 causing disulfide formation. This effect was further investigated by structure modeling, considering the structural similarity between NADP^+^ and ADP-glucose. The two main features distinguishing NADP^+^ from ADP-glucose are the phosphate group at the C2 position of adenosine ribose and the presence of C5-linked ribose-nicotinamide in place of C1-linked glucose. In our model, there is a preformed cavity in the *At*SS1 that can fit the C2-phosphate moiety without disturbing a binding mode analogous to that of ADP (S2 Fig c). Moreover, the nicotinamide group and Cys545 are in close proximity (S2 Fig d) and simple torsion angle rotations of the nicotinamide would allow contact. A putative covalent intermediate formed in this way could explain the incomplete recovery of activity after DTTred treatment of *At*SS1 incubated with high concentrations of NADP^+^ (S2 Fig b). The finding that inhibition by high concentrations of NADP^+^ is partially reversed by DTTred treatment suggests the presence also of a redox effect in the inhibition observed (S2 Fig b). An additional possible explanation of the reversibility test data (S2 Fig b) could be that NADP^+^ interferes with DTTred-mediated activation.
(DOCX)

S3 Fig SDS gels illustrating purification steps of 6xHis-*At*SS1 proteins emphasizing changed protein production in case of double C164S_C265S mutation. S, supernatant of centrifuged cell lysate; FT, flow-through; W1, washing step 1; W5, washing step 5 (the last one); E1-E6, elution fractions with increasing imidazole concentrations; M, marker. Marker size from the top arrow (kDa): 200, 116.3, 97.4, 66.3, 55.4, 36.5, 31, 21.5, 14.4, 6, 3.5, 2.5. In case of WT SDS gel, the second lane from the left contains a marker which was unintentionally mixed with the supernatant sample.
(DOCX)

S4 Fig Redox titrations at pH 7.9 of *At*SS1, four single mutants and one double mutant. The relationship between the redox potential and DTTred:DTTox concentration ratio was calculated using the Nernst equation. The titration data points were fitted by non-linear regression anticipating a two-electron transfer process for the thiol-disulfide to calculate midpoint potentials. The activity was assayed by SCGA using maltotriose as acceptor. Values are the mean of two independent experiments.
(DOCX)

S5 Fig The relative content of Cys residues in SS1 homologs. Cysteine content was calculated as a percentage ratio between cysteine residue number and total amino acid residue number. Red, *Dicotyledoneae* and *Monocotyledoneae*; orange, *Lycopodiophyta* and *Bryophyta*; olive, *Charophyta*; green, *Chlorophyta*; blue, *Bacteria*; purple, *Archaea*. Protein size and cysteine residue numbers are listed in S6 Table.
(DOCX)

S6 Fig *At*SS1 binding capacity to the maize starch granules under reduced and oxidized conditions. *At*SS1 protein (0.3 μg μL^-1^) was reduced with 20 mM DTTred or oxidized with 1 μM CuCl_2_ for 1h at 37°C. 4.2 mg of protein were incubated for 45 min at RT with a slow constant mixing using rotating wheel with 0, 20, 40, 60, 80, 100 or 200 mg mL^-1^ of native maize starch, BSA 0.05 mg mL^-1^ in a total volume of 350 μL of 20 mM TRIS buffer pH 8.0. Enzyme activity in the supernatant was assayed using the default protocol.
(DOCX)

S1 Protocol Protein sequences of *At*SS1, *At*Trx*f*1, *At*Trx*m*4, *At*NTRC. Primer sequences. Purification protocols for *At*SS1, *At*Trx*f*1, *At*Trx*m*4, *At*NTRC. (DOCX)

S1 Table Thermofluor stability of *At*SS1wild type protein treated with ADP (positive control), NADH, NADPH and NADP^+^. Results are expressed as differences in melting temperatures between treated and untreated (0 mM) samples. Thermostability of *At*SS1 was tested with a variant of the Thermofluor assay. The enzyme was diluted to 0.1 mg/mL in a buffer containing 20 mM Tris pH 8.0, 100 mM NaCl and 2X Sypro orange dye (Sigma-Aldrich, S5692). Various components were added to this buffer as specified in the results section. 50 μL of this solution were placed in individual wells of RT-PCR plates (MicroAmp® Optical 96-well reaction plate from Applied Biosystems, 4306737), sealed with adhesive film and centrifuged to remove bubbles and to create a flat surface. The plates were loaded in a 7500 RT-PCR system (Applied Biosystems) and subjected to a modified ramp protocol heating from 25°C to 95°C over 73 minutes (approx. 1°C min^-1^). Fluorescence was monitored with ROX filters and the minimum points of its derivative were read manually and interpreted as melting temperatures (Tm).
(DOCX)

S2 Table Activity and redox sensitivity of *At*SS1 wild type protein and cysteine-to-serine mutants analyzed by SCGA. Maltotriose was used as acceptor. Activity is expressed as enzyme turnovers min^-1^. Values are the mean of two technical replicates (±SD)
(DOCX)

S3 Table Local sequence alignment around KXGGL and ADP-glucose binding motifs. Conserved amino acid residues (grey), Cys residues (yellow). Alignment was done by tCOFFEE server (http://www.tcoffee.crg.cat/) and numbering based on the full-length protein sequence.
(DOCX)

S4 Table V_max_ and K_m_ towards ADP-glucose of reduced and oxidized forms of *At*SS1 wild type (WT) and mutant variants. Results are the mean of two independent experiments (±SD) and are expressed in turnovers of enzyme per minute. N.D. not determined.
(DOCX)

S5 Table Midpoint redox potentials of the *At*SS1 variants. The effect of the redox potential on *At*SS1 activity at pH 7.9 was determined by incubation in defined DTTred:DTTox ratios and subsequent activity assays. The activity was assayed by SCGA using maltotriose as acceptor. The titration data was fitted by non-linear regression using the Nernst equation anticipating a two-electron transfer process for the thiol-disulfide exchange reaction to calculate midpoint potentials. Values are the mean of two independent experiments (±SD).
(DOCX)

S6 Table Accession number from the NCBI or UniProt databases and taxonomic lineage of the organisms used in the study. (DOCX)
