# Supplementary material for: The Role of Cysteine Residues in Redox Regulation and Protein Stability of Arabidopsis thaliana Starch Synthase 1
Source: PLoS One. 2015 Sep 14;10(9):e0136997. doi: 10.1371/journal.pone.0136997 (PMC4569185; doi:10.1371/journal.pone.0136997)
Supplement: S1 Fig — (a) Overview of the redox reversibility experiment. (b) Results obtained by the activity assays. The AtSS1 protein was first treated with 0.3 mM dithiothreitol (DTTred) or 5 μM CuCl2 for reduction or oxidation of the enzyme, respectively. Following desalting, the pre-reduced protein was treated with 5 μM CuCl2 while the pre-oxidized protein was treated with DTTred (0.3 mM and 20 mM). The activity was assayed by SCGA using maltotriose as acceptor. The values represent the mean of two technical replicates (±SD). Red: reduced AtSS1, Ox: oxidized AtSS1. The activity of AtSS1 pre-reduced with reduced dithiothreitol (DTTred) decreased to 1.8% when treated with CuCl2. The untreated protein (no DTT) oxidized with CuCl2 had activity close to zero (0.3%) but activity could be restored to 67% after treatment with 0.3 mM DTTred. The apparent loss of AtSS1 activity following reactivation could be due to irreversible oxidation of redox-sensitive Cys residues. (DOCX) [file pone.0136997.s001.docx]

**Figure S1.**

**
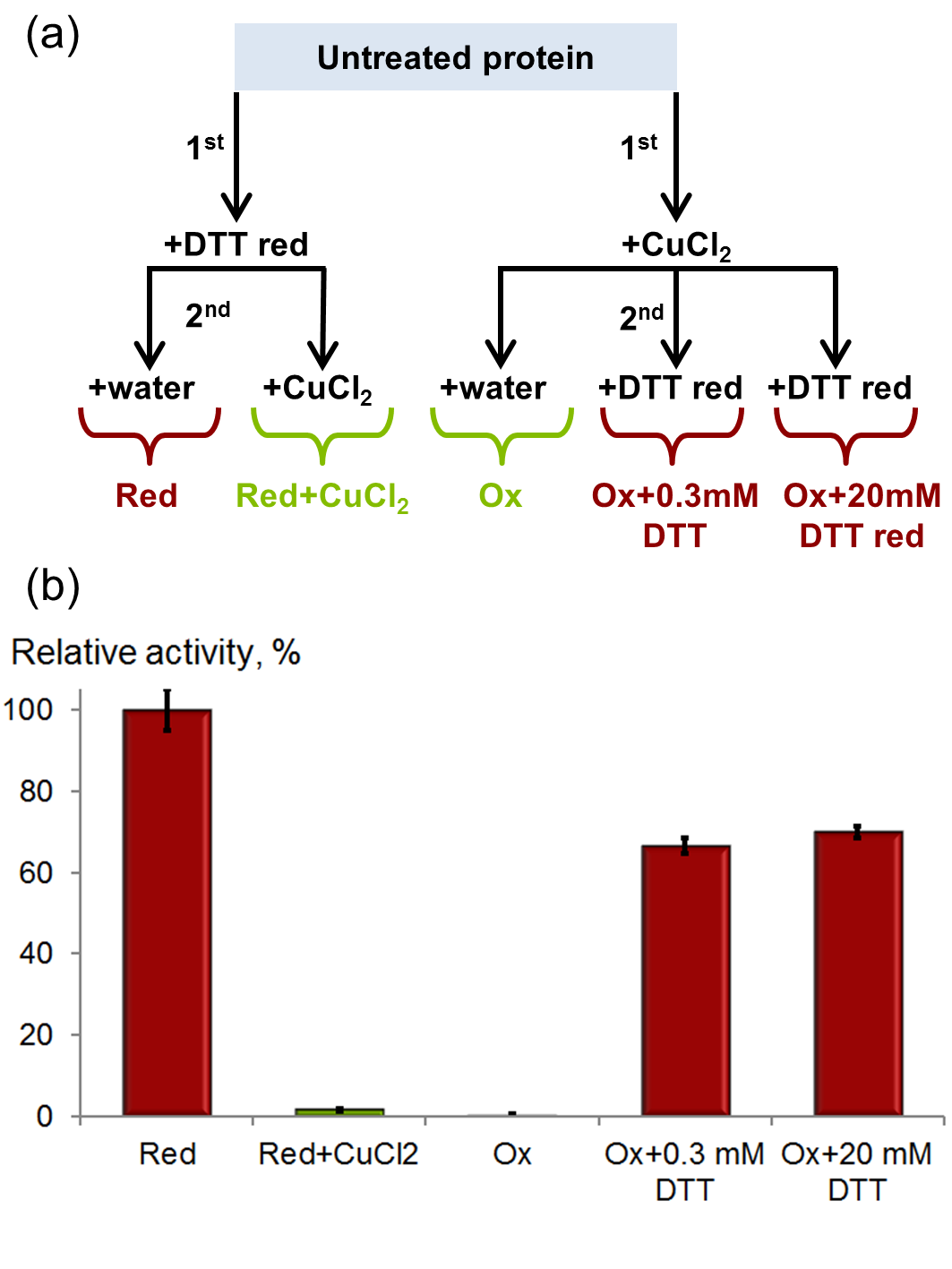
**

**Figure S1. Reversible redox modulation of recombinant *At*SS1 activity.**

(a) Overview of the redox reversibility experiment. (b) Results obtained by the activity assays. The *At*SS1 protein was first treated with 0.3 mM dithiothreitol (DTTred) or 5 µM CuCl_2_ for reduction or oxidation of the enzyme, respectively. Following desalting, the pre-reduced protein was treated with 5 µM CuCl_2_ while the pre-oxidized protein was treated with DTTred (0.3 mM and 20 mM). The activity was assayed by SCGA using maltotriose as acceptor. The values represent the mean of two technical replicates (±SD). Red: reduced *At*SS1, Ox: oxidized *At*SS1.

The activity of *At*SS1 pre-reduced with reduced dithiothreitol (DTTred) decreased to 1.8% when treated with CuCl_2_. The untreated protein (no DTT) oxidized with CuCl_2_ had activity close to zero (0.3%) but activity could be restored to 67% after treatment with 0.3 mM DTTred. The apparent loss of *At*SS1 activity following reactivation could be due to irreversible oxidation of redox-sensitive Cys residues.
