# Supplementary material for: The Role of Cysteine Residues in Redox Regulation and Protein Stability of Arabidopsis thaliana Starch Synthase 1
Source: PLoS One. 2015 Sep 14;10(9):e0136997. doi: 10.1371/journal.pone.0136997 (PMC4569185; doi:10.1371/journal.pone.0136997)
Supplement: S2 Fig — Enzyme activity was monitored by glycogen-containing NAG. (a) Activity of pre-reduced AtSS1 protein treated with 0, 0.1, 0.5, 1.0 and 10 mM NADP+ or 10 μM of CuCl2. (b) Activity of pre-reduced AtSS1 protein incubated with 2.5, 5, 7.5 and 10 mM NADP+, and incubated with either 1 mM ADP-glucose only (top gel) or 1 mM ADP-glucose supplemented with 20 mM DTTred (bottom gel). (c) Model of AtSS1 showing the active site area. Blue: the N-terminal domain of AtSS1, red: the C-terminal domain. Bound ADP-glucose (magenta) is represented by the ADP and glucose fragments unmodified from structure 2qzs [32] from the PDB. NADPH (green) has been modeled in a compatible conformation that avoids clashes with the protein. (d) Relative orientation of the nicotinamide moiety in the model (pink together with its ribose) and Cys545 (yellow). NADP+ was found to inhibit AtSS1 activity in vitro (S2 Fig a). Analysis of the concentration dependence of NADP+ on AtSS1 activity showed that the inhibition was reversible following incubation with 20 mM DTTred (S2 Fig b). To reveal a possible specific interaction between NADP+ and AtSS1, a ThermoFluor assay of protein stability was performed. The rationale behind this experiment was that any specific interaction might stabilize the protein as detected by an increased melting temperature [57]. The results (S1 Table) demonstrate elevated thermostability of AtSS1 protein pretreated with NADP+. This effect could be due to either direct binding of NADP+ or oxidation of AtSS1 causing disulfide formation. This effect was further investigated by structure modeling, considering the structural similarity between NADP+ and ADP-glucose. The two main features distinguishing NADP+ from ADP-glucose are the phosphate group at the C2 position of adenosine ribose and the presence of C5-linked ribose-nicotinamide in place of C1-linked glucose. In our model, there is a preformed cavity in the AtSS1 that can fit the C2-phosphate moiety without disturbing a binding mode [file pone.0136997.s002.docx]

**Figure S2.**

**
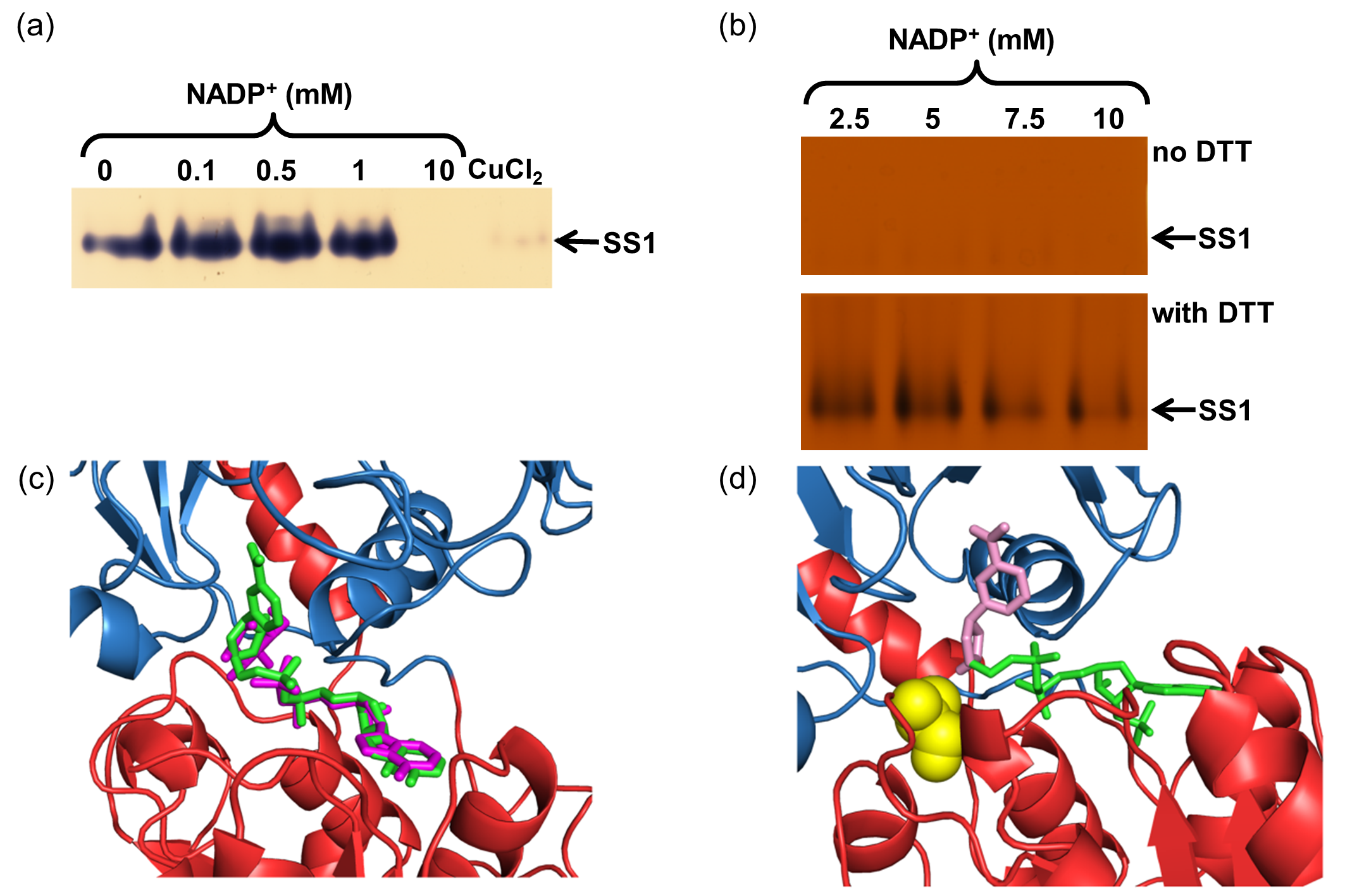
**

**Figure S2. Influence of NADP^+^ on activity of *At*SS1.**

Enzyme activity was monitored by glycogen-containing NAG. (a) Activity of pre-reduced *At*SS1 protein treated with 0, 0.1, 0.5, 1.0 and 10 mM NADP^+^ or 10 μM of CuCl_2_. (b) Activity of pre-reduced *At*SS1 protein incubated with 2.5, 5, 7.5 and 10 mM NADP^+^, and incubated with either 1 mM ADP-glucose only (top gel) or 1 mM ADP-glucose supplemented with 20 mM DTTred (bottom gel). (c) Model of *At*SS1 showing the active site area. Blue: the N-terminal domain of *At*SS1, red: the C-terminal domain. Bound ADP-glucose (magenta) is represented by the ADP and glucose fragments unmodified from structure 2qzs (1) from the PDB. NADPH (green) has been modeled in a compatible conformation that avoids clashes with the protein. (d) Relative orientation of the nicotinamide moiety in the model (pink together with its ribose) and Cys545 (yellow).

NADP^+^ was found to inhibit *At*SS1 activity *in vitro* (Fig. S2a). Analysis of the concentration dependence of NADP^+^ on *At*SS1 activity showed that the inhibition was reversible following incubation with 20 mM DTTred (Fig. S2b). To reveal a possible specific interaction between NADP^+^ and *At*SS1, a ThermoFluor assay of protein stability was performed. The rationale behind this experiment was that any specific interaction might stabilize the protein as detected by an increased melting temperature (2). The results (Table S1) demonstrate elevated thermostability of *At*SS1 protein pretreated with NADP^+^. This effect could be due to either direct binding of NADP^+^ or oxidation of *At*SS1 causing disulfide formation.

This effect was further investigated by structure modeling, considering the structural similarity between NADP^+^ and ADP-glucose. The two main features distinguishing NADP^+^ from ADP-glucose are the phosphate group at the C2 position of adenosine ribose and the presence of C5-linked ribose-nicotinamide in place of C1-linked glucose. In our model, there is a preformed cavity in the *At*SS1 that can fit the C2-phosphate moiety without disturbing a binding mode analogous to that of ADP (Fig. S2c). Moreover, the nicotinamide group and Cys545 are in close proximity (Fig. S2d) and simple torsion angle rotations of the nicotinamide would allow contact. A putative covalent intermediate formed in this way could explain the incomplete recovery of activity after DTTred treatment of *At*SS1 incubated with high concentrations of NADP^+^ (Fig. S2b).

The finding that inhibition by high concentrations of NADP^+^ is partially reversed by DTTred treatment suggests the presence also of a redox effect in the inhibition observed (Fig. S2b). An additional possible explanation of the reversibility test data (Fig. S2b) could be that NADP^+^ interferes with DTTred-mediated activation.
