# Supplementary material for: The Role of Cysteine Residues in Redox Regulation and Protein Stability of Arabidopsis thaliana Starch Synthase 1
Source: PLoS One. 2015 Sep 14;10(9):e0136997. doi: 10.1371/journal.pone.0136997 (PMC4569185; doi:10.1371/journal.pone.0136997)
Supplement: S3 Fig — S, supernatant of centrifuged cell lysate; FT, flow-through; W1, washing step 1; W5, washing step 5 (the last one); E1-E6, elution fractions with increasing imidazole concentrations; M, marker. Marker size from the top arrow (kDa): 200, 116.3, 97.4, 66.3, 55.4, 36.5, 31, 21.5, 14.4, 6, 3.5, 2.5. In case of WT SDS gel, the second lane from the left contains a marker which was unintentionally mixed with the supernatant sample. (DOCX) [file pone.0136997.s003.docx]

**Figure S3.**


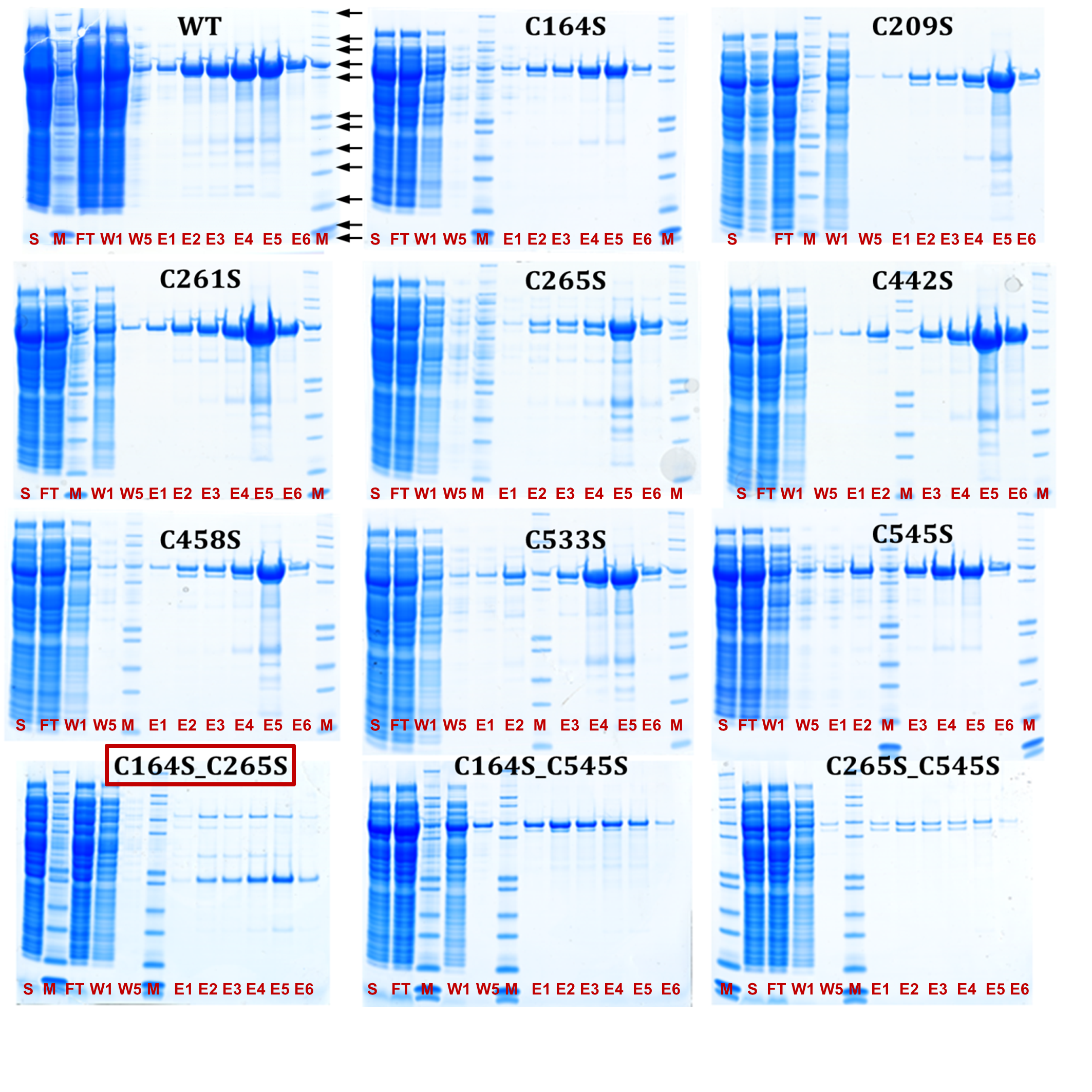


**Figure S3. SDS gels illustrating purification steps of 6xHis-*At*SS1 proteins emphasizing changed protein production in case of double C164S_C265S mutation.**

S, supernatant of centrifuged cell lysate; FT, flow-through; W1, washing step 1; W5, washing step 5 (the last one); E1-E6, elution fractions; M, marker. Marker size from the top arrow (kDa): 200, 116.3, 97.4, 66.3, 55.4, 36.5, 31, 21.5, 14.4, 6, 3.5, 2.5.
