# Supplementary material for: The Role of Cysteine Residues in Redox Regulation and Protein Stability of Arabidopsis thaliana Starch Synthase 1
Source: PLoS One. 2015 Sep 14;10(9):e0136997. doi: 10.1371/journal.pone.0136997 (PMC4569185; doi:10.1371/journal.pone.0136997)
Supplement: S4 Fig — The relationship between the redox potential and DTTred:DTTox concentration ratio was calculated using the Nernst equation. The titration data points were fitted by non-linear regression anticipating a two-electron transfer process for the thiol-disulfide to calculate midpoint potentials. The activity was assayed by SCGA using maltotriose as acceptor. Values are the mean of two independent experiments. (DOCX) [file pone.0136997.s004.docx]

**Figure S4.**


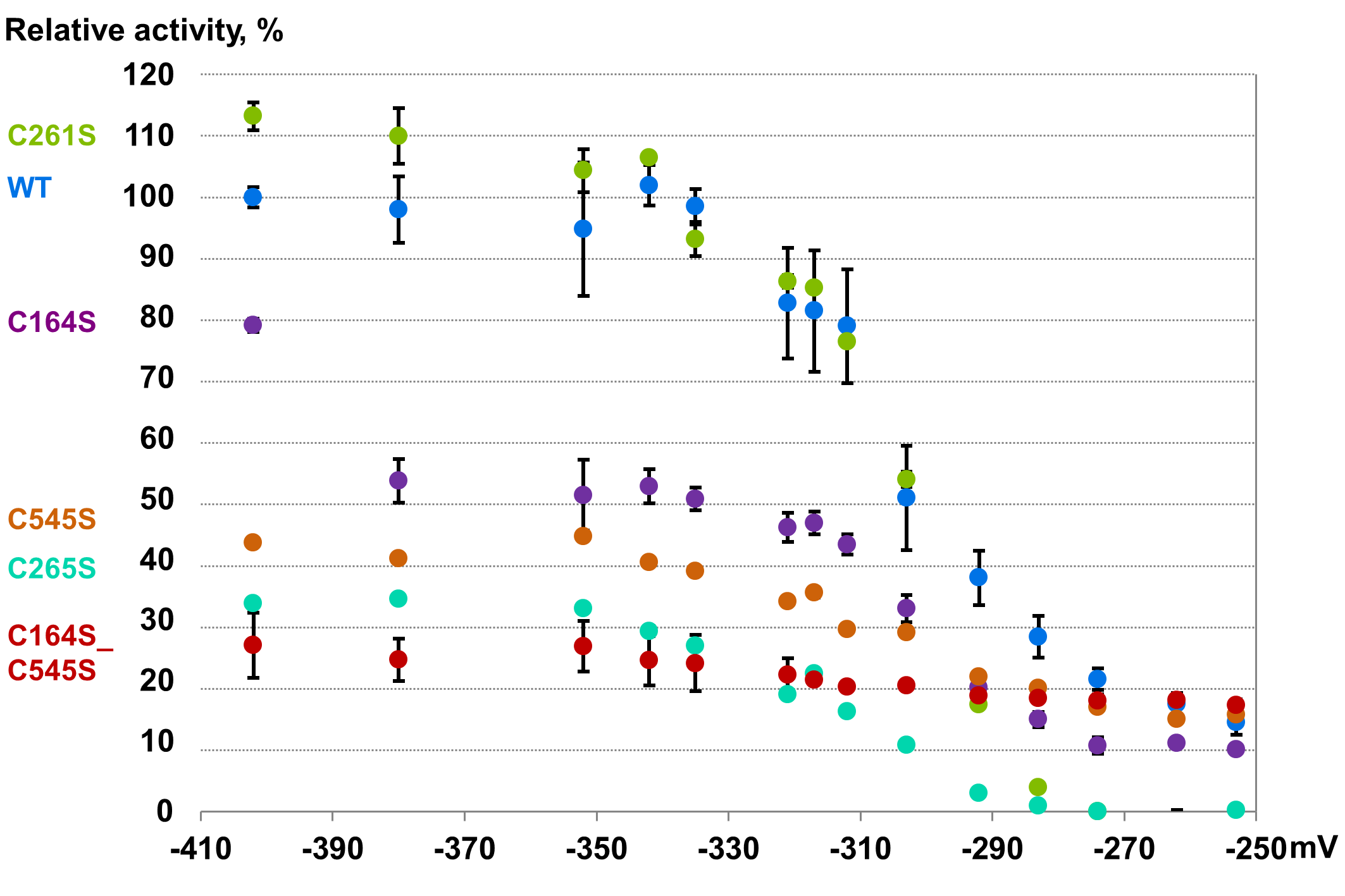


**Figure S4. Redox titrations at pH 7.9 of *At*SS1, four single mutants and one double mutant.**

The relationship between the redox potential and DTTred:DTTox concentration ratio was calculated using the Nernst equation. The titration data points were fitted by non-linear regression anticipating a two-electron transfer process for the thiol-disulfide to calculate midpoint potentials. The activity was assayed by SCGA using maltotriose as acceptor. Values are the mean of two independent experiments.
