# Supplementary material for: The Role of Cysteine Residues in Redox Regulation and Protein Stability of Arabidopsis thaliana Starch Synthase 1
Source: PLoS One. 2015 Sep 14;10(9):e0136997. doi: 10.1371/journal.pone.0136997 (PMC4569185; doi:10.1371/journal.pone.0136997)
Supplement: S5 Fig — Cysteine content was calculated as a percentage ratio between cysteine residue number and total amino acid residue number. Red, Dicotyledoneae and Monocotyledoneae; orange, Lycopodiophyta and Bryophyta; olive, Charophyta; green, Chlorophyta; blue, Bacteria; purple, Archaea. Protein size and cysteine residue numbers are listed in S6 Table. (DOCX) [file pone.0136997.s005.docx]

**Figure S5.**


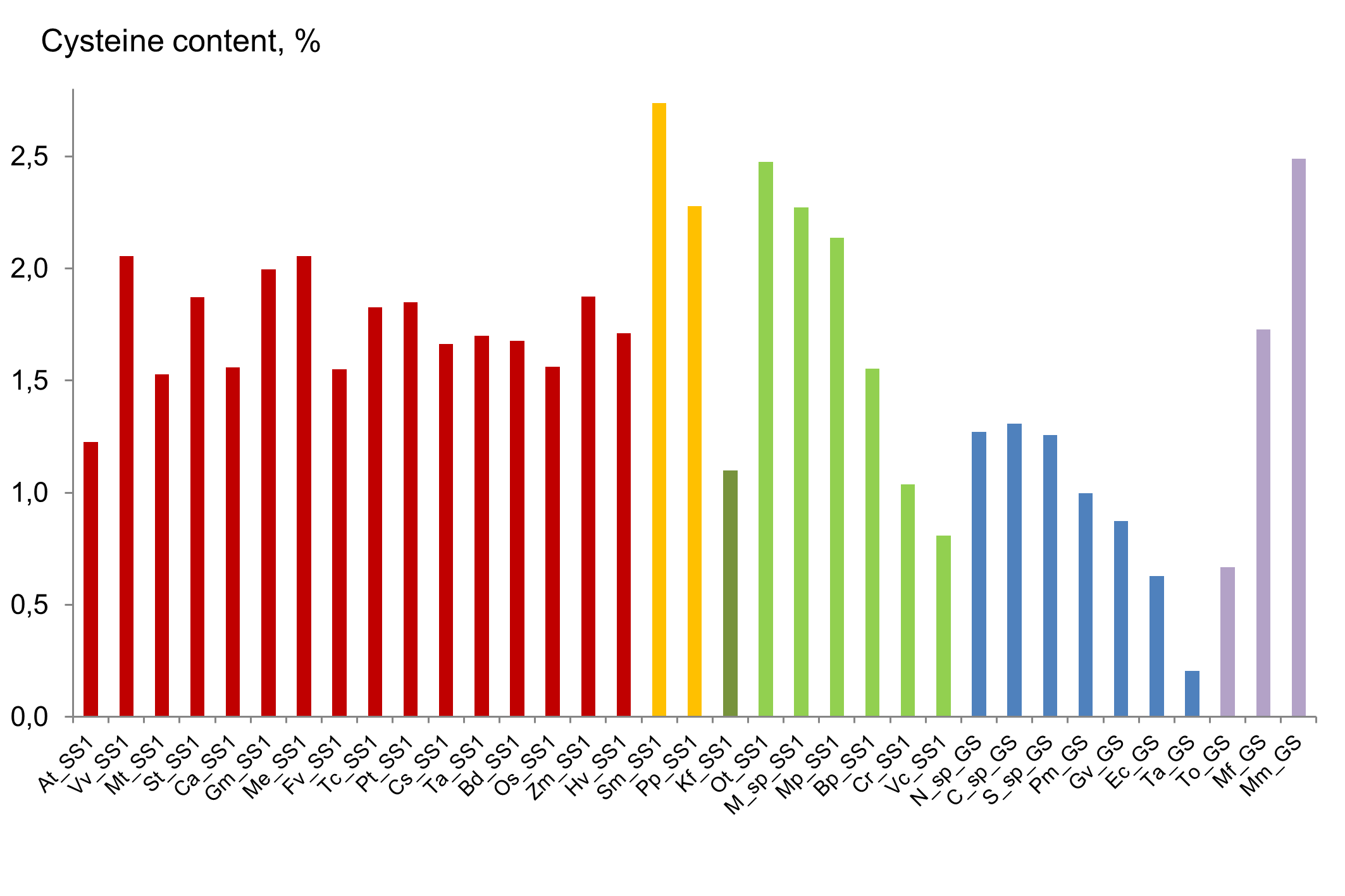


**Figure S5. The relative content of Cys residues in SS1 homologs.**

Cysteine content was calculated as a percentage ratio between cysteine residue number and total amino acid residue number. Red, *Dicotyledoneae* and *Monocotyledoneae*; orange, *Lycopodiophyta* and *Bryophyta*; olive, *Charophyta*; green, *Chlorophyta*; blue, *Bacteria*; purple, *Archaea*. Protein size and cysteine residue numbers are listed in Table S6.
