# Supplementary material for: The Role of Cysteine Residues in Redox Regulation and Protein Stability of Arabidopsis thaliana Starch Synthase 1
Source: PLoS One. 2015 Sep 14;10(9):e0136997. doi: 10.1371/journal.pone.0136997 (PMC4569185; doi:10.1371/journal.pone.0136997)
Supplement: S6 Fig — AtSS1 protein (0.3 μg μL-1) was reduced with 20 mM DTTred or oxidized with 1 μM CuCl2 for 1h at 37°C. 4.2 mg of protein were incubated for 45 min at RT with a slow constant mixing using rotating wheel with 0, 20, 40, 60, 80, 100 or 200 mg mL-1 of native maize starch, BSA 0.05 mg mL-1 in a total volume of 350 μL of 20 mM TRIS buffer pH 8.0. Enzyme activity in the supernatant was assayed using the default protocol. (DOCX) [file pone.0136997.s006.docx]

Figure S6.


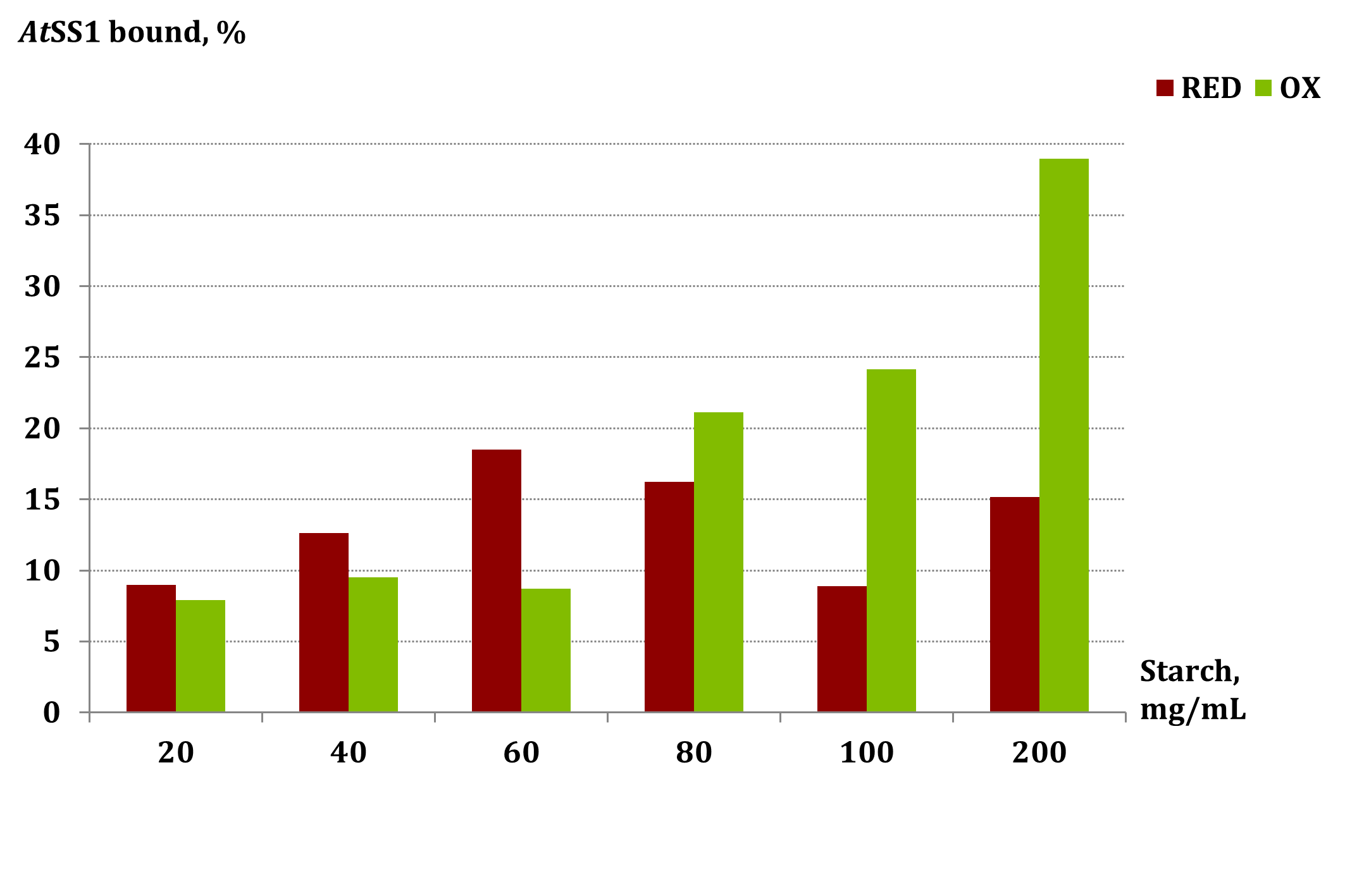


**Figure S6. *At*SS1 binding capacity to the maize starch granules under reduced and oxidized conditions.**

*At*SS1 protein (0.3 µg µL^-^1) was reduced with 20 mM DTTred or oxidized with 1 µM CuCl_2_ for 1h at 37 °C. 4.2 mg of protein were incubated for 45 min at RT with a slow constant mixing using rotating wheel with 0, 20, 40, 60, 80, 100 or 200 mg mL^-1^ of native maize starch, BSA 0.05 mg mL^-1^ in a total volume of 350 µL of 20 mM TRIS buffer pH 8.0. Enzyme activity in the supernatant was assayed using the default protocol.
