# Supplementary material for: The Role of Cysteine Residues in Redox Regulation and Protein Stability of Arabidopsis thaliana Starch Synthase 1
Source: PLoS One. 2015 Sep 14;10(9):e0136997. doi: 10.1371/journal.pone.0136997 (PMC4569185; doi:10.1371/journal.pone.0136997)
Supplement: S1 Protocol — (DOCX) [file pone.0136997.s007.docx]

**Protocol S1. Protein sequences of *At*SS1, *At*Trx*f*1, *At*Trx*m*4, *At*NTRC. Primer sequences. Purification protocols for *At*SS1, *At*Trx*f*1, *At*Trx*m*4, *At*NTRC.**

**Protein sequence of recombinant *At*SS1**

MGSSHHHHHHSSGLVPRGSHMASSSSFSGDSRESDEERFITDAERDGSGSVLGFQLTPPGDQQTVSTSTGEITHHEEKKEAIDQIVMADFGVPGNRAVEEGAAEVGIPSGKAEVVNNLVFVTSEAAPYSKTGGLGDVCGSLPIALAGRGHRVMVISPRYLNGTAADKNYARAKDLGIRVTVNCFGGSQEVSFYHEYRDGVDWVFVDHKSYHRPGNPYGDSKGAFGDNQFRFTLLCHAACEAPLVLPLGGFTYGEKSLFLVNDWHAGLVPILLAAKYRPYGVYKDARSILIIHNLAHQGVEPAATYTNLGLPSEWYGAVGWVFPTWARTHALDTGEAVNVLKGAIVTSDRIITVSQGYAWEITTVEGGYGLQDLLSSRKSVINGITNGINVDEWNPSTDEHIPFHYSADDVSEKIKCKMALQKELGLPIRPECPMIGFIGRLDYQKGIDLIQTAGPDLMVDDIQFVMLGSGDPKYESWMRSMEETYRDKFRGWVGFNVPISHRITAGCDILLMPSRFEPCGLNQLYAMRYGTIPVVHGTGGLRDTVENFNPYAEGGAGTGTGWVFTPLSKDSMVSALRLAAATYREYKQSWEGLMRRGMTRNYSWENAAVQYEQVFQWVFMDPPYVS

*At*SS1 wild type MW=68790 Da, single mutant *At*SS1 MW=68774 Da, double mutant *At*SS1 MW=68758 Da. ε_280_ = 1,624 M^-1^ cm^-1^ for wild type protein and 1,625 M^-1^ cm^-1^ for mutated protein.

**Primers used for site directed mutagenesis for cysteine to serine substitution**

Target triplets are indicated in lowercase letters.

| **CYS** | **Forward primer** | **Reverse primer** |
| --- | --- | --- |
| **164** | CGGTCTGGGTGATGTGtcaGGTTCTCTGCCGATTGCC | GGCAATCGGCAGAGAACCtgaCACATCACCCAGACCG |
| **209** | CATTCGCGTCACGGTGAATagtTTTGGCGGTTCCCAAG | CTTGGGAACCGCCAAAactATTCACCGTGACGCGAATG |
| **261** | CACAAGCTGCATGGCtcaGCAGCGTGAAACG | CGTTTCACGCTGCtgaGCCATGCAGCTTGTG |
| **265** | TGTGCCATGCAGCTagtGAAGCACCGCTG | CAGCGGTGCTTCactAGCTGCATGGCACA |
| **442** | GCTGATGACGTTAGTGAAAAAAtcaAAAGCAAAATGGCGCTG | CAGCGCCATTTTGCTTTtgaTTTTTTCACTAACGTCATCAGC |
| **458** | GCCGATCCGTCCGGAAagtCCGATGATTG | CAATCATCGGactTTCCGGACGGATCGGC |
| **533** | CGTATCACCGCCGGTagcGACATCCTGC | GCAGGATGTCgctACCGGCGGTGATACG |
| **545** | CGTCACGCTTTGAACCGtctGGCCTGAATCAGCTGTAC | GTACAGCTGATTCAGGCCagaCGGTTCAAAGCGTGACG |

**Primers used for sequencing *At*SS1**

| **Primer sequence** | **Mutation verified** |
| --- | --- |
| GGAACAGACTTTTTTCACCATAGG (reverse primer) | C164S, C209S |
| TCCCAAGAAGTCTCATTCTATCAC (forward primer 1) | C261S, C265S, C442S |
| AACATATCCCGTTCCACTACTCTG (forward primer 2) | C458S, C533S, C545S |

**Protein sequence of recombinant *At*Trx*f*1** (MW=15400 Da. ε_280_ = 1,102)

MGSSHHHHHHSSGLVPRGSHMLETVNVSVGQVTEVDKDTFWPIVKAAGEKLVVLDMYTQWCGPCKVIAPKYKALSEKYDDVVFLKLDCNPDNRPLAKELGIRVVPTFKILKDNKVVKEVTGAKYDDLVAAIETARSAASG

**Protein sequence of recombinant *At*Trx*m*4** (MW=15440 Da. ε_280_ = 1,262)

MGSSHHHHHHSSGLVPRGSHMEAQDTTAAAVEVPNLSDSEWQTKVLESDVPVLVEFWAPWCGPCRMIHPIVDQLAKDFAGKFKFYKINTDESPNTANRYGIRSVPTVIIFKGGEKKDSIIGAVPRETLEKTIERFLVE

**Protein sequence of recombinant *At*NTRC** (MW=53437 Da. ε_280_ = 0,839; ε_450_ = 11,300 M^-1^ cm^-1^)

MSYYHHHHHHLESTSLYKKAAATANSPSSSSSGGEIIENVVIIGSGPAGYTAAIYAARANLKPVVFEGYQMGGVPGGQLMTTTEVENFPGFPDGITGPDLMEKMRKQAERWGAELYPEDVESLSVTTAPFTVQTSERKVKCHSIIYATGATARRLRLPREEEFWSRGISACAICDGASPLFKGQVLAVVGGGDTATEEALYLTKYARHVHLLVRRDQLRASKAMQDRVINNPNITVHYNTETVDVLSNTKGQMSGILLRRLDTGEETELEAKGLFYGIGHSPNSQLLEGQVELDSSGYVLVREGTSNTSVEGVFAAGDVQDHEWRQAVTAAGSGCIAALSAERYLTSNNLLVEFHQPQTEEAKKEFTQRDVQEKFDITLTKHKGQYALRKLYHESPRVILVLYTSPTCGPCRTLKPILNKVVDEYNHDVHFVEIDIEEDQEIAEAAGIMGTPCVQFFKNKEMLRTISGVKMKKEYREFIEANK

***At*SS1 production and purification**

*At*SS1 proteins of wild type and mutants were expressed as N-terminally 6xHis-tagged polypeptides in *E. coli* Tuner (DE3) cells (Novagen). Pre-cultures from freshly transformed cells were grown over night (37 °C, 30 µg ml^-1^ kanamycin in LB-broth Miller medium - Formedium, LMM0102) and then added to the main culture flask (0.3-0.5% of the final volume) together with kanamycin (30 µg ml^-1^) and one drop of antifoam (Sigma, A6426). The volume of the main culture was kept 10-20% of the total flask volume; baffled flasks were used for better aeration. At OD_600_ 0.6 the culture was cooled on ice for 10 min and expression induced with 100 mM isopropyl-β-D-1-thiogalactopyranoside (IPTG) for 20 h (16 °C, 160 rpm shaking rate). Alternatively, a protocol using auto induction medium (Formedium, AIMLB0210) not requiring induction with IPTG was applied. For this protocol flasks were incubated for 2.5 h at 37 °C following 24 h incubation at 18 °C. Cells were harvested by 30 min centrifugation at 3200 g and 4 °C. Cell pellets were resuspended in ice cold buffer A (20 mM TRIS pH 8.0, 0.5 M NaCl, 20 mM imidazole, 10% (v/v) glycerol) supplemented with protease inhibitor tablets (1 per 50 ml, Roche, 05056489001), 10 mM MgSO_4_, one drop of antifoam, DNAse I (4 µg/ml, Roche, 10104159001). The resuspended pellets were disrupted by using a one shot cell disrupter (Constant System Ltd.) at 1.35 kBar. Disrupted cells were centrifuged for 1 h at 48 000 g at 4 °C and the cell lysate was filtered through a 0.22 µM syringe filter. Proteins were affinity purified using either Nickel Magnetic Agarose Beads (NMAB, Sigma, H9914) applied on filter collection column (Evergreen, 208-3049-03S) or HisTrap columns (GEHealthcare) connected to an ÄKTA FPLC (Pharmacia). In the first case a stepwise elution (40, 60 and 500 mM imidazole; centrifugation at 50 g) was applied, in the second case proteins were eluted with a gradient 0-250 mM imidazole. Based on SDS gels fractions containing pure protein were pooled together and dialyzed against 20 mM TRIS pH 8.0, 0.2 M NaCl, 10% (v/v) glycerol. The dialyzed proteins were aliquoted and stored at -80 °C and -20 °C.

**Production and purification of recombinant Trxs**

The open reading frames of *A. thaliana* thioredoxins *f*1 (AT3G02730) and *m*4 (AT3G15360), excluding the predicted chloroplast transit peptide (amino acids 1-59 and 1-76 for *f*1 and *m*4, respectively), were synthesized as codon-optimized constructs for expression in *E. coli*. The ORFs were cloned directly into the expression vector pET15b and expressed as N-terminally 6xHistagged proteins in *E. coli* BL21 (DE3). After OD_600_ was 0.6 expression was induced by adding 1mM IPTG and cells were harvested after 4 h of incubation at 30 °C. Cells were disrupted by one shot cell disrupter (Constant System Ltd.) at 1.35 kBar in the buffer containing 50 mM Tris pH 8.0, 300 mM NaCl, 20 mM imidazole supplemented with protease inhibitor tablets, 10 mM MgSO_4_, one drop of antifoam, DNAse I (4 µg/ml, Roche, 10104159001). Proteins were purified using 1 ml HisTrap columns (GE Healthcare) connected to an ÄKTA FPLC (Pharmacia). The bound thioredoxins was washed with 50 mM Tris pH 8.0, 300 mM NaCl, 20 mM imidazole, 0.5% v/v Triton and eluted with a stepwise gradient of imidazole in elution buffer (50 mM Tris pH 8.0, 50 mM NaCl, 50-500 mM imidazole) and stored at -80 °C in 50 mM Tris pH 8.0, 10% (v/v) glycerol.

**Production and purification of recombinant NTRC protein**

*Arabidopsis thaliana* NTRC (UniProtKB/Swiss-Prot: O22229.2) was expressed in *E. coli* Rosetta2 (DE3) transformed with the pDEST17 expression vector containing an insert with the mature NTRC transcript. Cells were grown in LB medium with 34 µg ml^-1^ chloramphenicol and 100 µg ml^-1^ ampicillin until an OD_600_ of 0.5 and then induced with 1 mM IPTG. Expression was allowed to proceed overnight with shaking at 20 °C. Cells were harvested by centrifugation at 10 000 g for 20 min, resuspended in cold lysis buffer (30 mM Tris pH 8.0, 500 mM NaCl, 20 mM imidazole) supplemented with protease inhibitor tablets and broken with a TS series benchtop cell disruptor (Constant Systems Ltd) using two passes at 35 kPsi. Lysate was cleared by centrifugation at 16 000 g for 20 min at 4 °C, and loaded on a HisTrap HP column (GE Healthcare) connected to an ÄKTA FPLC (Pharmacia). Elution was achieved using a stepwise gradient of 50 mM and 150 mM imidazole. Yellow fractions were pooled, concentrated using Amicon centrifugal filters (Millipore) with a cutoff of 30 kDa and injected on a HiLoad 26/60 Superdex 200 gel filtration column (GE Healthcare) in 30 mM Tris pH 8.0 with 150 mM NaCl. Fractions from the major NTRC-containing peak were pooled, concentrated as above and stored in aliquots at -80 °C. Activity was verified by monitoring the reduction of DTNB to TNB^-­^ spectrophotometrically at 412 nm (ε_412_ = 14,150 M^-1^ cm^-1^) in a reaction mixture containing 30 mM Tris pH 8, 30 mM NaCl, 0.57 nmole *At*NTRC, 32 µM DTNB (Sigma) and 160 µM NADPH (Sigma).
