# Supplementary material for: The Role of Cysteine Residues in Redox Regulation and Protein Stability of Arabidopsis thaliana Starch Synthase 1
Source: PLoS One. 2015 Sep 14;10(9):e0136997. doi: 10.1371/journal.pone.0136997 (PMC4569185; doi:10.1371/journal.pone.0136997)
Supplement: S1 Table — Results are expressed as differences in melting temperatures between treated and untreated (0 mM) samples. Thermostability of AtSS1 was tested with a variant of the Thermofluor assay. The enzyme was diluted to 0.1 mg/mL in a buffer containing 20 mM Tris pH 8.0, 100 mM NaCl and 2X Sypro orange dye (Sigma-Aldrich, S5692). Various components were added to this buffer as specified in the results section. 50 μL of this solution were placed in individual wells of RT-PCR plates (MicroAmp® Optical 96-well reaction plate from Applied Biosystems, 4306737), sealed with adhesive film and centrifuged to remove bubbles and to create a flat surface. The plates were loaded in a 7500 RT-PCR system (Applied Biosystems) and subjected to a modified ramp protocol heating from 25°C to 95°C over 73 minutes (approx. 1°C min-1). Fluorescence was monitored with ROX filters and the minimum points of its derivative were read manually and interpreted as melting temperatures (Tm). (DOCX) [file pone.0136997.s008.docx]

**Table S1. Thermofluor stability of *At*SS1 wild type protein treated with ADP (positive control), NADH, NADPH and NADP^+^.**

Results are expressed as differences in melting temperatures between treated and untreated (0 mM) samples. Thermostability of *At*SS1 was tested with a variant of the Thermofluor assay. The enzyme was diluted to 0.1 mg/mL in a buffer containing 20 mM Tris pH 8.0, 100 mM NaCl and 2X Sypro orange dye (Sigma-Aldrich, S5692). Various components were added to this buffer as specified in the results section. 50 μL of this solution were placed in individual wells of RT-PCR plates (MicroAmp® Optical 96-well reaction plate from Applied Biosystems, 4306737), sealed with adhesive film and centrifuged to remove bubbles and to create a flat surface. The plates were loaded in a 7500 RT-PCR system (Applied Biosystems) and subjected to a modified ramp protocol heating from 25 °C to 95 °C over 73 minutes (approx. 1 °C min^-1^). Fluorescence was monitored with ROX filters and the minimum points of its derivative were read manually and interpreted as melting temperatures (Tm).

| **Compound** | **0 mM** | **0.5 mM** | **1 mM** | **2 mM** | **3 mM** | **4 mM** | |
| --- | --- | --- | --- | --- | --- | --- | --- |
| **ADP** | 0 | 0.5 | 0.7 | 1.4 | 1.4 | 2.4 | |
| **NADH** | 0 | 0 | 0 | 0.1 | 0.3 | 0.1 | |
| **NADPH** | 0 | 0 | 0 | -0.1 | -0.1 | -0.3 | |
| **NADP^+^** | 0 | 0 | 0.2 | 0 | 0.4 | 0.6 | |
| **Compound** | **0 mM** | **1 mM** | **3 mM** | **6 mM** | **10 mM** | **20 mM** |  |
| **NADPH** | 0 | 0.2 | 0.2 | 0 | 0.2 | -0.1 |  |
| **NADP^+^** | 0 | 0.2 | 0.4 | 0.7 | 0.9 | 1.5 |  |
