# Supplementary material for: The Role of Cysteine Residues in Redox Regulation and Protein Stability of Arabidopsis thaliana Starch Synthase 1
Source: PLoS One. 2015 Sep 14;10(9):e0136997. doi: 10.1371/journal.pone.0136997 (PMC4569185; doi:10.1371/journal.pone.0136997)
Supplement: S2 Table — Maltotriose was used as acceptor. Activity is expressed as enzyme turnovers min-1. Values are the mean of two technical replicates (±SD) (DOCX) [file pone.0136997.s009.docx]

**Table S2. Activity and redox sensitivity of *At*SS1 wild type protein and cysteine-to-serine mutants analyzed by SCGA.**

Maltotriose was used as acceptor. Activity is expressed as enzyme turnovers min^-1^. Values are the mean of two technical replicates (±SD).

| **Protein** | **Reduced form** | | **Oxidized form** | |
| --- | --- | --- | --- | --- |
|  | **Absolute activity A_red_** | **Relative activity (%)** | **Absolute activity A_ox_** | **Redox sensitivity (%)** |
| **WT** | 146.6±2.4 | 100 | 33.7±0.2 | 77 |
| **C164S** | 73.6±2.4 | 50 | 15.0±0.1 | 80 |
| **C209S** | 145.3±4.9 | 99 | 1.3±0.0 | 99 |
| **C261S** | 120.1±0.8 | 82 | 1.4±0.2 | 99 |
| **C265S** | 54.2±0.2 | 37 | 1.3±0.2 | 98 |
| **C442S** | 139.3±0.5 | 95 | 1.1±0.1 | 99 |
| **C458S** | 136.8±1.2 | 93 | 1.6±0.2 | 99 |
| **C533S** | 155.2±5.7 | 106 | 1.5±0.0 | 99 |
| **C545S** | 40.3±0.9 | 27 | 18.0±0.0 | 55 |
| **C164S_C265S** | 6.5±0.0 | 4 | 2.7±0.3 | 59 |
| **C164S_C545S** | 18.5±0.5 | 13 | 13.0±0.2 | 30 |
| **C265S_C545S** | 11.3±0.3 | 8 | 3.5±0.1 | 69 |
