# Supplementary material for: The Role of Cysteine Residues in Redox Regulation and Protein Stability of Arabidopsis thaliana Starch Synthase 1
Source: PLoS One. 2015 Sep 14;10(9):e0136997. doi: 10.1371/journal.pone.0136997 (PMC4569185; doi:10.1371/journal.pone.0136997)
Supplement: S3 Table — Conserved amino acid residues (grey), Cys residues (yellow). Alignment was done by tCOFFEE server (http://www.tcoffee.crg.cat/) and numbering based on the full-length protein sequence. (DOCX) [file pone.0136997.s010.docx]

**Table S3. Local sequence alignment around KXGGL and ADP-glucose binding motifs.**

Conserved amino acid residues (grey), Cys residues (yellow). Alignment was done by tCOFFEE server (http://www.tcoffee.crg.cat/) and numbering based on the full-length protein sequence.

| **GS*/*SSs** | **AA No** | **KXGGL motif** | **AA No** | **ADP-glucose binding motif** |
| --- | --- | --- | --- | --- |
| ***At*SS1** | 156 | KTGGLGDVCGSL | 539 | PSRFEPCGLNQLYAMRYGTIP |
| ***Ec*GS** | 15 | KTGGLADVIGAL | 373 | PSRFEPCGLTQLYGLKYGTLP |
| ***Hv*SS1** | 149 | KSGGLGDVCGSL | 531 | PSRFEPCGLNQLYAMQYGTVP |
| ***At*GBSS** | 98 | KTGGLGDVLGGL | 482 | PSRFEPCGLIQLHAMRYGTVP |
| ***At*SS2** | 315 | KTGGLGDVAGAL | 687 | PSRFEPCGLNQLYAMNYGTIP |
| ***At*SS3** | 591 | KVGGLGDVVTSL | 915 | PSIFEPCGLTQLIAMRYGAVP |
| ***At*SS4** | 556 | KVGGLGDVVAGL | 925 | PSIFEPCGLTQMIAMRYGSIP |
