# Supplementary material for: The Role of Cysteine Residues in Redox Regulation and Protein Stability of Arabidopsis thaliana Starch Synthase 1
Source: PLoS One. 2015 Sep 14;10(9):e0136997. doi: 10.1371/journal.pone.0136997 (PMC4569185; doi:10.1371/journal.pone.0136997)
Supplement: S4 Table — Results are the mean of two independent experiments (±SD) and are expressed in turnovers of enzyme per minute. N.D. not determined. (DOCX) [file pone.0136997.s011.docx]

**Table S4. V_max_ and K_m_ towards ADP-glucose of reduced and oxidized forms of *At*SS1 wild type (WT) and mutant variants.**

Results are the mean of two independent experiments (±SD) and are expressed in turnovers of enzyme per minute. N.D. not determined.

| **Protein** | **Reduced** | | | | **Oxidized** | | | |
| --- | --- | --- | --- | --- | --- | --- | --- | --- |
|  | **V_max_** | | **K_m_** | | **V_max_** | | **K_m_** | |
|  | **Absolute** | **Relative to WT V_max_ (%)** | **Absolute** | **Relative to WT reduced K_m_** | **Absolute** | **Redox sensitivity (%)** | **Absolute** | **Relative to WT reduced K_m_** |
| **WT** | 192±3 | 100 | 0.076±0.005 | 1.00 | 31.9±0.5 | 83 | 0.053±0.004 | 0.70 |
| **C164S** | 69.5±1.4 | 36 | 0.102±0.007 | 1.33 | 13.8±0.3 | 80 | 0.096±0.007 | 1.3 |
| **C209S** | 172±4 | 90 | 0.127±0.009 | 1.67 | N.D. | N.D. | N.D. | N.D. |
| **C261S** | 183±4 | 95 | 0.185±0.012 | 2.44 | 1.3±0.1 | 99 | 0.076±0.029 | 1.00 |
| **C265S** | 24.6±1.1 | 13 | 0.073±0.014 | 0.96 | N.D. | N.D. | N.D. | N.D. |
| **C442S** | 164±3 | 86 | 0.098±0.006 | 1.28 | N.D. | N.D. | N.D. | N.D. |
| **C458S** | 168±3 | 88 | 0.101±0.006 | 1.32 | 2.0±0.2 | 99 | 0.331±0.086 | 4.4 |
| **C533S** | 177±3 | 93 | 0.107±0.007 | 1.41 | 2.8±0.2 | 98 | 0.132±0.035 | 1.7 |
| **C545S** | 73.8±2.0 | 38 | 0.74±0.07 | 9.7 | 27.6±0.4 | 63 | 0.733±0.032 | 9.6 |
| **C164S_C265S** | 8.9±0.6 | 5 | 0.32±0.06 | 4.22 | N.D. | N.D. | N.D. | N.D. |
| **C164S_C545S** | 54.0±1.9 | 28 | 1.22±0.12 | 16.0 | 40.0±0.9 | 26 | 1.49±0.084 | 19.5 |
| **C265S_C545S** | 16.3±0.4 | 9 | 0.84±0.04 | 11.0 | N.D. | N.D. | N.D. | N.D. |
