# Supplementary material for: The Role of Cysteine Residues in Redox Regulation and Protein Stability of Arabidopsis thaliana Starch Synthase 1
Source: PLoS One. 2015 Sep 14;10(9):e0136997. doi: 10.1371/journal.pone.0136997 (PMC4569185; doi:10.1371/journal.pone.0136997)
Supplement: S5 Table — The effect of the redox potential on AtSS1 activity at pH 7.9 was determined by incubation in defined DTTred:DTTox ratios and subsequent activity assays. The activity was assayed by SCGA using maltotriose as acceptor. The titration data was fitted by non-linear regression using the Nernst equation anticipating a two-electron transfer process for the thiol-disulfide exchange reaction to calculate midpoint potentials. Values are the mean of two independent experiments (±SD). (DOCX) [file pone.0136997.s012.docx]

**Table S5. Midpoint redox potentials of the *At*SS1 variants.**

The effect of the redox potential on *At*SS1 activity at pH 7.9 was determined by incubation in defined DTTred:DTTox ratios and subsequent activity assays. The activity was assayed by SCGA using maltotriose as acceptor. The titration data was fitted by non-linear regression using the Nernst equation anticipating a two-electron transfer process for the thiol-disulfide exchange reaction to calculate midpoint potentials. Values are the mean of two independent experiments (±SD).

| **Protein** | **Midpoint redox potentials** |
| --- | --- |
| **WT** | -306 mV |
| **C164S** | -300 mV |
| **C261S** | -304 mV |
| **C265S** | -313 mV |
| **C545S** | -308 mV |
