# Supplementary material for: The Role of Cysteine Residues in Redox Regulation and Protein Stability of Arabidopsis thaliana Starch Synthase 1
Source: PLoS One. 2015 Sep 14;10(9):e0136997. doi: 10.1371/journal.pone.0136997 (PMC4569185; doi:10.1371/journal.pone.0136997)
Supplement: S6 Table — (DOCX) [file pone.0136997.s013.docx]

**Table S6. Accession number from the NCBI or UniProt databases and taxonomic lineage of the organisms used in the study.**

| **Protein name_\|AccNo\|**  **aa No/Cys No** | **Organism name** | **Taxonomic lineage (from UniProt; http://www.uniprot.org/)** |
| --- | --- | --- |
| **At_SS1_\|Q9FNF2\| 652/8** | *Arabidopsis thaliana* | Eukaryota>Viridiplantae>Streptophyta>Embryophyta>Tracheophyta>Spermatophyta>Magnoliophyta>Eudicotyledons>Coreeudicotyledons>Rosids>Malvids>Brassicales>Brassicaceae>Camelineae>Arabidopsis |
| **Vv_SS1_\|F6H795\| 633/13** | *Vitis vinifera* | Eukaryota>Viridiplantae>Streptophyta>Embryophyta>Tracheophyta>Spermatophyta>Magnoliophyta>  [Eudicotyledons](http://www.uniprot.org/taxonomy/71240)>[Coreeudicotyledons](http://www.uniprot.org/taxonomy/91827)>[Rosids](http://www.uniprot.org/taxonomy/71275)>[Vitales](http://www.uniprot.org/taxonomy/403667)>[Vitaceae](http://www.uniprot.org/taxonomy/3602)>[Vitis](http://www.uniprot.org/taxonomy/3603) |
| **Mt_SS1_\|G7J7E9\| 655/10** | *Medicago truncatula* | Eukaryota>Viridiplantae>Streptophyta>Embryophyta>Tracheophyta>Spermatophyta>Magnoliophyta>  [Eudicotyledons](http://www.uniprot.org/taxonomy/71240)>Coreeudicotyledons>Rosids>[Fabids](http://www.uniprot.org/taxonomy/91835)>[Fabales](http://www.uniprot.org/taxonomy/72025)>[Fabaceae](http://www.uniprot.org/taxonomy/3803)>[Papilionoideae](http://www.uniprot.org/taxonomy/3814)>[Trifolieae](http://www.uniprot.org/taxonomy/163742)>[Medicago](http://www.uniprot.org/taxonomy/3877) |
| **St_SS1_\|P93568\| 641/12** | *Solanum tuberosum* | Eukaryota>Viridiplantae>Streptophyta>Embryophyta>Tracheophyta>Spermatophyta>Magnoliophyta>  [Eudicotyledons](http://www.uniprot.org/taxonomy/71240)>[Coreeudicotyledons](http://www.uniprot.org/taxonomy/91827)>[Asterids](http://www.uniprot.org/taxonomy/71274)>[Lamiids](http://www.uniprot.org/taxonomy/91888)>[Solanales](http://www.uniprot.org/taxonomy/4069)>[Solanaceae](http://www.uniprot.org/taxonomy/4070)>[Solanoideae](http://www.uniprot.org/taxonomy/424551)>[Solaneae](http://www.uniprot.org/taxonomy/424574)>[Solanum](http://www.uniprot.org/taxonomy/4107) |
| **Ca_SS1_\|XP_004502657.1\| 642/10** | *Cicer arietinum* | Eukaryota>Viridiplantae>Streptophyta>Embryophyta>Tracheophyta>Spermatophyta>Magnoliophyta>  Eudicotyledons>Gunneridae>Pentapetalae>Rosids>Fabids>Fabales>Fabaceae>Papilionoideae>Cicereae>Cicer |
| **Gm_SS1_\|I1JYR0\| 651/13** | *Glycine max* | Eukaryota>Viridiplantae>Streptophyta>Embryophyta>Tracheophyta>Spermatophyta>Magnoliophyta>Eudicotyledons>Coreeudicotyledons>Rosids>Fabids>Fabales>Fabaceae>Papilionoideae>Phaseoleae>Glycine |
| **Me_SS1_\|B3SRP2\| 633/13** | *Manihot esculenta* | Eukaryota>Viridiplantae>Streptophyta>Embryophyta>Tracheophyta>Spermatophyta>Magnoliophyta>Eudicotyledons>Coreeudicotyledons>Rosids>Fabids>Malpighiales>Euphorbiaceae>Crotonoideae>Manihoteae>Manihot |
| **Fv_SS1_\|XP_004307998.1\| 645/10** | *Fragaria vesca subsp. vesca* | Eukaryota>Viridiplantae>Streptophyta>Embryophyta>Tracheophyta>Spermatophyta>Magnoliophyta>Eudicotyledons>Gunneridae>Pentapetalae>Rosids>Fabids>Rosales>Rosaceae>Rosoideae>Potentilleae>Fragariinae>  [Fragaria](http://www.uniprot.org/taxonomy/3746) |
| **Tc_SS1_\|EOY23571.1\| 657/12** | *Theobroma cacao* | Eukaryota>Viridiplantae>Streptophyta>Embryophyta>Tracheophyta>Spermatophyta>Magnoliophyta>Eudicotyledons>Gunneridae>Pentapetalae>Rosids>Malvids>Malvales>Malvaceae>Byttnerioideae>Theobroma |
| **Pt_SS1_\|B9IDQ2\|649/12** | *Populus trichocarpa* | Eukaryota>Viridiplantae>Streptophyta>Embryophyta>Tracheophyta>Spermatophyta>Magnoliophyta>Eudicotyledons>Coreeudicotyledons>Rosids>Fabids>Malpighiales>Salicaceae>Saliceae>Populus |
| **Cs_SS1_\|XP_006493328.1\| 662/11** | *Citrus sinensis* | Eukaryota>Viridiplantae>Streptophyta>Embryophyta>Tracheophyta>Spermatophyta>Magnoliophyta>Eudicotyledons>Gunneridae>Pentapetalae>Rosids>Malvids>Sapindales>Rutaceae>Citrus |
| **Ta_SS1_\|Q9LEC0\| 647/11** | *Triticum aestivum* | Eukaryota>Viridiplantae>Streptophyta>Embryophyta>Tracheophyta>Spermatophyta>Magnoliophyta>Liliopsida>Poales>Poaceae>BEP Clade>Pooideae>Triticeae>Triticum |
| **Bd_SS1_\|I1H0T2\| 656/11** | *Brachypodium distachyon* | Eukaryota>Viridiplantae>Streptophyta>Embryophyta>Tracheophyta>Spermatophyta>Magnoliophyta>Liliopsida>Poales>Poaceae>BEP Clade>Pooideae>Brachypodieae>Brachypodium |
| **Os_SS1_\|A2Y9M4\| 641/10** | *Oryza sativa subsp. indica* | Eukaryota>Viridiplantae>Streptophyta>Embryophyta>Tracheophyta>Spermatophyta>Magnoliophyta>Liliopsida>Poales>Poaceae>BEP Clade>Ehrhartoideae>Oryzeae>Oryza |
| **Zm_SS1_\|O49064\| 640/12** | *Zea mays* | Eukaryota>Viridiplantae>Streptophyta>Embryophyta>Tracheophyta>Spermatophyta>Magnoliophyta>Liliopsida>Poales>Poaceae>PACMAD clade>Panicoideae>Andropogoneae>Zea |
| **Hv_SS1_\|Q9M5A3\| 643/11** | *Hordeum vulgare* | Eukaryota>Viridiplantae>Streptophyta>Embryophyta>Tracheophyta>Spermatophyta>Magnoliophyta>Liliopsida>Poales>Poaceae>BEP Clade>Pooideae>Triticeae>Hordeum |
| **Sm_SS1_\|D8QWI4\| 548/15** | *Selaginella moellendorffii* | Eukaryota>Viridiplantae>Streptophyta>Embryophyta>Tracheophyta>Lycopodiophyta>Isoetopsida>  [Selaginellales](http://www.uniprot.org/taxonomy/3244)>[Selaginellaceae](http://www.uniprot.org/taxonomy/3245)>[Selaginella](http://www.uniprot.org/taxonomy/3246) |
| **Pp_SS1_\|A9U0N5\| 527/12** | *Physcomitrella patens subsp. patens* | Eukaryota>Viridiplantae>Streptophyta>Embryophyta>Bryophyta>Bryophytina>Bryopsida>  [Funariidae](http://www.uniprot.org/taxonomy/114656)>[Funariales](http://www.uniprot.org/taxonomy/3215)>[Funariaceae](http://www.uniprot.org/taxonomy/3216)>[Physcomitrella](http://www.uniprot.org/taxonomy/3217) |
| **Kf_SS1_\|kfl00793_0010\| 731/8** | *Klebsormidium flaccidum* | Eukaryota>Viridiplantae>Streptophyta>Klebsormidiophyceae>Klebsormidiales>Klebsormidiaceae>  [Klebsormidium](http://www.uniprot.org/taxonomy/3174) |
| **Ot_SS1_\|Q6PYY4\| 525/13** | *Ostreococcus tauri* | Eukaryota>Viridiplantae>Chlorophyta>Mamiellophyceae>Mamiellales>Ostreococcus |
| **M_SS1A_\|C1E8R9\| 572/13** | *Micromonas sp.* | Eukaryota>Viridiplantae>Chlorophyta>Mamiellophyceae>Mamiellales>Micromonas |
| **Mp_SS1A_\|C1MT67\| 702/15** | *Micromonas pusilla* | Eukaryota>Viridiplantae>Chlorophyta>Mamiellophyceae>Mamiellales>Micromonas |
| **Bp_SS1_\|K8FDF4\| 708/11** | *Bathycoccus prasinos* | Eukaryota>Viridiplantae>Chlorophyta>Mamiellophyceae>Mamiellales>Bathycoccus |
| **Cr_SS1_\|A8J9R4\| 579/6** | *Chlamydomonas reinhardtii* | Eukaryota>Viridiplantae>Chlorophyta>Chlorophyceae>Chlamydomonadales>Chlamydomonadaceae>  [Chlamydomonas](http://www.uniprot.org/taxonomy/3052) |
| **Vc_SS1_\|D8UHG9\| 1115/9** | *Volvox carteri* | Eukaryota>Viridiplantae>Chlorophyta>Chlorophyceae>Chlamydomonadales>Volvocaceae>Volvox |
| **N_GS_A1_\|Q8YVU5\| 472/6** | *Nostoc sp.* | Bacteria>Cyanobacteria>[Nostocales](http://www.uniprot.org/taxonomy/1161)>[Nostocaceae](http://www.uniprot.org/taxonomy/1162)>[Nostoc](http://www.uniprot.org/taxonomy/1177) |
| **C_GS_\| V5SQ38\| 459/6** | *Cyanobacterium sp. CLg1* | Bacteria>Cyanobacteria>Oscillatoriophycideae>Chroococcales>Cyanobacterium |
| **S_GS_\|P74521\| 477/6** | *Synechocystis sp.* | Bacteria>Cyanobacteria>Oscillatoriophycideae>Chroococcales>Synechocystis |
| **Pm_GS_\|Q7VBP0\| 501/5** | *Prochlorococcus marinus* | Bacteria>Cyanobacteria>[Prochlorales](http://www.uniprot.org/taxonomy/1212)>[Prochlorococcaceae](http://www.uniprot.org/taxonomy/1217)>[Prochlorococcus](http://www.uniprot.org/taxonomy/1218) |
| **Gv_GS_\|Q7NM37\| 458/4** | *Gloeobacter violaceus* | Bacteria>Cyanobacteria>[Gloeobacteria](http://www.uniprot.org/taxonomy/307596)>[Gloeobacterales](http://www.uniprot.org/taxonomy/307595)>[Gloeobacter](http://www.uniprot.org/taxonomy/33071) |
| **Ec_GS_A1\|Q1R5J7\| 477/3** | *Escherichia coli* | Bacteria>Proteobacteria>Gammaproteobacteria>Enterobacteriales>Enterobacteriaceae>Escherichia |
| **Ta_GS_A_\|B7IFD0\| 485/1** | *Thermosipho africanus* | Bacteria>[Thermotogae](http://www.uniprot.org/taxonomy/200918)>[Thermotogales](http://www.uniprot.org/taxonomy/2419)>[Thermotogaceae](http://www.uniprot.org/taxonomy/188709)>[Thermosipho](http://www.uniprot.org/taxonomy/2420) |
| **To_GS_\|B6YV60\| 450/3** | *Thermococcus onnurineus* | Archaea>Euryarchaeota>Thermococci>Thermococcales>Thermococcaceae>Thermococcus |
| **Mf_GS_\|C7P5E0\| 521/9** | *Methanocaldococcus fervens* | Archaea>Euryarchaeota>Methanococci>Methanococcales>Methanocaldococcaceae>Methanocaldococcus |
| **Mm_GS_\|Q6LXQ5\| 522/13** | *Methanococcus maripaludis* | Archaea>Euryarchaeota>Methanococci>Methanococcales>Methanococcaceae>Methanococcus |
